# Signaling Pathways and Natural Compounds in Triple-Negative Breast Cancer Cell Line

**DOI:** 10.3390/molecules27123661

**Published:** 2022-06-07

**Authors:** Citra Dewi, Adryan Fristiohady, Riezki Amalia, Nur Kusaira Khairul Ikram, Sugeng Ibrahim, Muchtaridi Muchtaridi

**Affiliations:** 1 Department of Pharmaceutical Analysis and Medicinal Chemistry, Faculty of Pharmacy, Universitas Padjadjaran, Sumedang 45363, Indonesia; citra18005@mail.unpad.ac.id; 2 Pharmacy Department, Faculty of Science and Technology, Mandala Waluya University, Kendari 93561, Indonesia; 3 Faculty of Pharmacy, Halu Oleo University, Kampus Hijau Bumi Tridharma, Kendari 93232, Indonesia; adryanfristiohady@uho.ac.id; 4 Department of Pharmacology and Clinical Pharmacy, Faculty of Pharmacy, Universitas Padjadjaran, Sumedang 45363, Indonesia; riezki.amalia@unpad.ac.id; 5 Institute of Biological Sciences, Faculty of Science, Universiti Malaya, Kuala Lumpur 50603, Malaysia; nkusaira@um.edu.my; 6 Department of Molecular Biology, Faculty of Medicine, Universitas Katolik Soegijapranata, Semarang 50234, Indonesia; sugeng@unika.ac.id

**Keywords:** triple-negative breast cancer (TNBC), cell lines inhibitors, natural compounds

## Abstract

Triple-negative breast cancer (TNBC) is the most aggressive subtype of breast cancer, having a poor prognosis and rapid metastases. TNBC is characterized by the absence of estrogen, progesterone, and human epidermal growth receptor-2 (HER2) expressions and has a five-year survival rate. Compared to other breast cancer subtypes, TNBC patients only respond to conventional chemotherapies, and even then, with limited success. Shortages of chemotherapeutic medication can lead to resistance, pressured index therapy, non-selectivity, and severe adverse effects. Finding targeted treatments for TNBC is difficult owing to the various features of cancer. Hence, identifying the most effective molecular targets in TNBC pathogenesis is essential for predicting response to targeted therapies and preventing TNBC cell metastases. Nowadays, natural compounds have gained attention as TNBC treatments, and have offered new strategies for solving drug resistance. Here, we report a systematic review using the database from Pubmed, Science Direct, MDPI, BioScince, Springer, and Nature for articles screening from 2003 to 2022. This review analyzes relevant signaling pathways and the prospect of utilizing natural compounds as a therapeutic agent to improve TNBC treatments in the future.

## 1. Introduction

According to the Globocan Database of the International Agency for Research on Cancer (IARC), breast cancer had the highest incidence in 2018. Breast cancer mortality rates in Indonesia were about 16.7%, or 58.256 million, while morbidity rates were around 11%, or 22.692 million. The majority of triple-negative breast cancer (TNBC) patients are young women with a BRCA1 gene mutation [1]. TNBC is characterized by the absence of estrogen, progesterone, and HER2 receptor expression [2]. It comprises about 15–20% of all breast cancer cases [3].

The major cause of mortality in TNBC patients is metastasis [4] to distant areas such as the bone, lung, and brain, rather than the tumor of breast cancer [5,6]. The metastatic migration or spread of breast cancer from primary tumors to other cell components is initiated by intravasation, survival, extravasation in circulation, and colonization [7]. Tumor cell instability can also potentially induce metastasis, allowing these cells to spread to other tissues [8]. TNBC patients have a dismal prognosis and cannot be treated with endocrine treatment or HER2-targeted therapies [9]. Consequently, this type of metastasis breast cancer requires special treatment approaches [10].

TNBC is usually treated using traditional methods such as surgery, radiation therapy, and chemotherapy [11]. Based on previous studies, chemotherapy administered before surgery indicates a favorable pathology response and a high survival rate [12]. By analyzing tissue from a cancer cell that had been presented in a surgical procedure but was not active, the efficiency of neoadjuvant chemotherapy was able to be determined [13]. It is widely recognized as a substantial advantage, although it can also lead to resistance [14,15,16]. As a result, patients with TNBC who receive untargeted treatment have a poor prognosis, requiring the development of novel breast cancer treatments such as anti-cancer and anti-metastasis medicines [9].

A majority of the studies focus on cancer therapeutics derived from natural substances, primarily phytochemicals [17]. Phytochemicals are natural compounds that either directly influence particular molecular targets such as genes or indirectly affect metabolic pathways by stabilizing the conjugates [18]. With their ability to induce epithelial–mesenchymal transition (EMT), apoptosis, and metastasis, phytochemicals could be a potential molecular targeted therapy, involving some signaling pathways, such as Wnt/β-Catenin [19], NF-κB [20], PI3K/Akt/mTOR [21], PD-1/PD-L1 [22], LAG-3 [23], CTLA-4 [24], STAT-3 [25], EGFR [26], Trop-2 [27], RAF/MEK/ERK [28], JAK [29], Glycoprotein NMB (GpNMB) [30], and Hedgehog [31]. Furthermore, several studies have demonstrated that a variety of natural compounds, such as luteolin, curcumin, α-mangostin, chalcones, piperin, fisetin, quercetin, resveratrol, silibinine, apigenin, genistein, 10-gingerol, berberine, epigallocatechin gallate, cyanidin-3-o-glucoside, and glycyrrhizin, have anti-cancer activity and may be employed as a therapeutic strategy through various mechanisms [32,33,34,35]. Therefore, natural compounds have gained attention and importance as anti-cancer agents owing to their safety, fewer adverse side effects, and ability to reduce chemotherapeutic drug resistance. They also improve antiproliferative effects and efficacy in targeting multiple signaling pathways in cancers, including TNBCs [36]. This review focuses on TNBC and their relevant signaling pathways, as well as the various bioactive natural compounds derived from plants that have a potential inhibitory effect against TNBC.

## 2. Methods

This review was made based on the results of the collection and review of journals obtained from the Pubmed, Science Direct, MDPI, BioScince, Springer, and Nature databases, with several related keywords such as “TNBC AND natural compounds AND TNBC Subtype”, “TNBC mechanism AND natural compounds AND antiTNBC”, “TNBC agent therapy AND natural compounds AND TNBC molecular”, “signaling pathways AND natural compounds”, “target therapy of TNBC AND signaling pathways”, “TNBC treatments AND natural compounds AND clinical study”, “TNBC AND cell lines inhibitors AND clinical study”, “cell lines AND natural compounds AND TNBC”, and “TNBC classification AND clinical study AND anti TNBC”.

The inclusion criteria for the main article are articles published in ≥2016 and research articles that discuss the mechanism of molecular pathways of triple-negative breast cancer and the regulation mechanism of phytochemicals on triple-negative breast cancers. Inclusion criteria for supporting articles are articles that discuss biomarkers or biological subtypes of triple-negative breast cancer in treatment strategies for triple-negative breast cancer subtypes. This supporting article is taken from articles published between 2003–2022, with most of the articles included being published after 2016. Exclusion criteria for the main articles were not related to natural compounds associated with TNBC.

**Figure 1:**
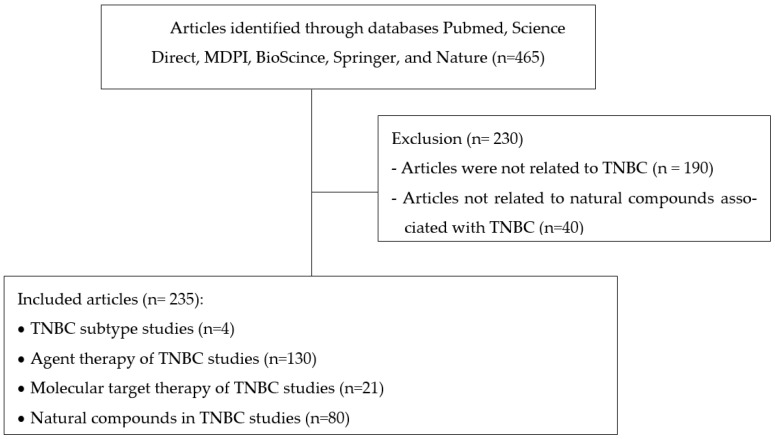
Article literature search flow chart.

This systematic review collected 465 publications from Pubmed, Science Direct, MDPI, BioScince, Springer, and Nature from 2003 to 2022. However, 230 were excluded, with 190 articles not related to TNBC and 40 articles not related to natural compounds associated with TNBC. After the first screening, 5 review papers were eliminated, yielding 235 articles containing, 4 TNBC subtype studies, 130 agent therapy of TNBC studies, 21 molecular target therapy of TNBC studies, and 80 natural compounds in TNBC studies. The article search flow can be seen in Figure 1.

## 3. Triple-Negative Breast Cancer

Triple-negative breast cancer (TNBC), accounts for about 10–15% of all breast cancer cases, and this is due to the lack of immunohistological expression of progesterone receptor (PR), estrogen receptor (ER), and human epidermal growth factor receptor 2 (HER2). This disease is characterized as a malignant tumor that is invasive and susceptible to the first metastasis [11]. TNBC has been associated with differential breast cancer, which is still difficult to characterize in the molecular phase due to the lack of a definitive prognostic marker and specific targeted therapy. Moreover, TNBC is indicated as a type of breast cancer that has an aggressive clinical behavior, a high rate of proliferation, and a poor prognosis, as well as a mutation in the breast cancer gene 1 (BRCA1) [37,38,39].

### 3.1. Classification of Triple-Negative Breast

TNBC gene expression is heterogeneous, and six subtypes have been identified based on molecular analysis: basal-like (BL-1 and BL-2), immunomodulatory (IM), mesenchymal-like (M), mesenchymal stem-like (MSL), and luminal androgen receptors (LAR). In independent research, Masuda et al. (2013) categorized TNBC into seven subtypes with strong associations (BL1, BL2, M, IM, MSN, LAR), one of which is an unstable subtype (UNS).

The BL-1 subtype has the highest prevalence of TP53 gene mutations, which affects gene expression, cell cycle, DNA damage response, and regulation. In contrast, the BL-2 subtype was associated with high gene expression of the growth factor pathway and metabolic pathway activity. The IM subtype is related to the immune cell pathway, high antigen, and cytokine signaling expression including TNF, NF-κB, and JAK/STAT pathways. The mesenchymal and MSL subtypes are responsible for gene expression to cell motility, cellular differentiation, and epithelial-mesenchymal transition (EMT) in the MSL of angiogenesis-enriched genes, while the LAR subtype is enriched for androgen receptor expression and has higher mutation genes in PI3KCA, AKT1, and CDH1. The intrinsic basal-like subtype was seen in many BL-1 and BL-2 cancers associated with BRCA mutations [40]. The molecular classification of TNBC and ongoing clinical potential therapies in vitro is shown in Table 1.

Samples from 14 datasets of extracted 374 TNBC were collected to establish the connection between the TNBC subtypes and intrinsic molecular (PAM50) subtypes. Most TNBC samples are categorized as basal-like (80.6%), using PAM50 subtype, followed by HER2 (38.10%), normal-like (17.5%), luminal B (13.3%), and luminal A (4,1%) [46,47].

Six TNBC molecular clusters were identified by two in silico studies. Basal-like 1, basal-like 2, immunomodulatory, mesenchymal, mesenchymal stem-like, and luminal androgen receptors were discovered in the first study, while immunity 1, immunity 2, proliferation/DNA damage, androgen receptor-like, matrix/invasion 1, and matrix/invasion 2 were described in the subsequent study [48].

### 3.2. Targeted Therapy of Triple-Negative Breast Cancer

**Figure 2:**
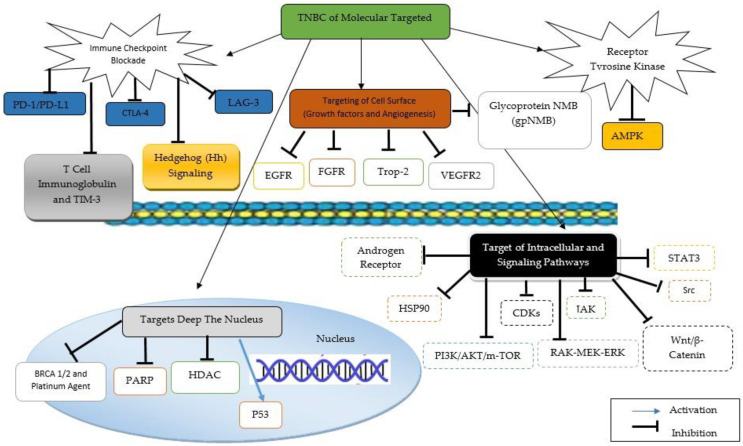
Mechanism of targeted therapies in TNBC.

Various efforts have been carried out to examine the problems in TNBC treatment. Chemotherapy, such as anthracyclines, ixabepilone, taxanes, and platinum drugs, is the most common treatment for TNBC patients [49]. However, not all chemotherapy patients had beneficial results, and it is still unclear whether the treatment is based on their TNBC subtypes. Efforts in developing therapies for target-specific TNBC biomarkers and TNBC therapy are ongoing [50]. These strategies, which include EGFR-targeted agents, androgen receptor-targeted agents, anti-antigenic agents, PARP inhibitors, immune-targeted, and Wnt/β-catenin signaling pathways, provide options for the triple-negative disease. However, their use in clinical trials is limited, and more research is needed to identify targets with high therapeutic ratios [51]. The mechanism of targeted therapies in TNBC is shown in Figure 2.

#### 3.2.1. Immune Checkpoint Blockade

##### Programmed Cell Death Protein 1 (PD-1) and Programmed Death-Ligand 1 (PDL-1)

The progress of immunotherapy in breast cancer is related to the biological nature of breast cancer and the immune system. Cancer is caused by a variety of processes that avoid the reaction of the immune system. Activated T-cells, pro-B cells, natural killer cells, dendritic cells, and monocytes all express the PD-1 antigen [52]. PD-1 and its ligands, PD-L1 and PD-L2, have a significant role in maintaining T-cell tolerance [52,53]. PD-1 and PD-L1 are explicitly expressed in basal-like breast cancer [53].

##### Cytotoxic T Lymphocyte-Associated Protein 4 (CTLA-4)

CTLA-4 is a type 1 receptor expressed in lymphocytes and T-cells with an IgV-like domain. When CTLA-4 is activated, it is found in intracellular vesicles and is quickly exported to the cell surface, resulting in efficient regulatory T-cell (Treg) suppression [52]. CTLA-4 is one of the immune checkpoint proteins expressed on activated T-cells [54]. The current study has led researchers to believe that utilizing Tregs as an anti-CTLA-4 therapy is one of the most critical factors for therapeutic responses [55,56].

CD28 is a protein constructor for CTLA-4. Both ligands, CD80 and CD86, are identical, but CTL-4 has a greater affinity. CD28 and CTLA-4 also have the same intracellular bonding pairs, the tp85 subunit of PI3K and PPA2 phosphatase. CTLA-4 is also expressed on regulatory T-cells mediating immunosuppressive responses. CTLA-4 suppresses T-cells by binding to CD80 and CD86, preventing CD28 stimulation and inhibiting T-cell activation. Another way is through CTLA-4 depleting B7 protein in APC, preventing B7 from performing its critical function of suppressing immunological responses in the body [57]. Ipilimumab is a checkpoint blocker and an anti-CTLA-4 monoclonal antibody that is presently being tested in clinical trials in combination with nivolumab or the combination of nivolumab and INCAGN01876 (anti-human glucocorticoid-induced tumor necrosis factor [TNF] receptor). Tremelimumab, an anti-CTLA-4 monoclonal antibody, is being tested in combination with PF-06936308, nab-paclitaxel, Carboplatin, and durvalumab in clinical trials.

##### Lymphocyte Activation Gene 3 (LAG-3)

LAG-3 is a type 1 transmembrane protein with CD4-like properties. LAG-3 has an immune system suppressive effect, although the exact mechanism is uncertain. As previously mentioned, LAG-3 has a larger extracellular domain than other immune checkpoint molecules, and its intracellular mechanisms are unique from those of other immune checkpoints. Expressions of LAG-3 have been detected in activated T cells, B cells, NK cells, and plasmacytoid dendritic cells. LAG-3 binds to MHC II receptors with a higher affinity level. Antigen-presenting cells are amplified with a competitive inhibitor on LAG-3/MHC II receptor binding. When combined with paclitaxel, IMP321 (LAG-3Ig) had an objective response rate of 50% as the first-line therapy for TNBC [58].

##### T Cell Immunoglobulin and Mucin-Domain Containing-3 (TIM-3)

TIM-3 is a member of the TIM protein family and an immunological checkpoint that works in conjunction with PD-1 and LAG-3 to weaken CD8+ T cells. Immune cells including monocytes and macrophages, dendritic cells, mast cells, and natural killer cells all express TIM-3. In addition, TIM-3 mediates the stimulation of the T-cel-CD8 response. INCAGN02390, an anti-TIM-3 antibody, is currently undergoing phase I clinical trials in a variety of advanced malignancies, including TNBC [59].

##### Hedgehog (Hh) Signaling Pathway

The Hh signaling pathway is involved in angiogenesis, embryogenesis, and cell fate regulation. This signaling pathway regulates the immune system and has been linked to TNBC growth and cancer cell stemness. In TNBC relationships with low overall survival, the hedgehog ligand has a noble expression.

TNBC cells grow, invade, and migrate more quickly when the Hh pathway is activated [31,60]. Three glioma-associated oncogenes (GLI) transcription factors, GLI1, GLI2, and GLI3, are effectors of Hh signaling that regulate the expression of pathway target genes [61]. TNBC has higher basal expression levels of the Hh signaling pathway gene such as GLI1 and GLI2, which are downstream of Hh ligands, than other breast cancers [31].

According to preclinical research, the Hh pathway plays a key role in the maintenance of the cancer stem cell phenotype, activation of cancer-associated fibroblasts, invasive behavior, and angiogenesis in TNBC. The activation mechanism is mostly non-canonical, including direct transcriptional upregulation of GLI1 and GLI2. The United States Food and Drug Administration (FDA) has approved two Hh signaling inhibitors, Vismodegib (NCT02694224) and sonidegib (NCT02027376), for clinical studies in TNBC patients.

Extrinsic regulation was obtained by upregulating GLI1 transcription in Hh signaling pathway activation, such as the PI3K-Akt-mTOR pathway [62], K-Ras, c-Myc, Wnt-beta catenin, and TGFβ [31,63,64]. Deviating transcriptional upregulation of GLI1 is seen downstream of NF-κB in claudin-low breast cancer, a sub-type of TNBC [65]. Furthermore, NF-B induced the transcription factor Forkhead Box C1 (FOXC1), which is an upstream mediator of Hh signaling via upregulation of GLI2 expression in basal-like breast cancer cells. In TNBC cell lines, inhibitors of the Hh pathway, such as GANT61 and Thiostrepton, were shown to inhibit stem cell phenotypes including CD44+/CD24ve cells and sphere-forming capacity [31].

#### 3.2.2. Target Deep the Nucleus

##### Breast Cancer Susceptibility Gene (BRCA) and Platinum-Based Treatment

BRCA1 and BRCA2 are two different tumor suppressor genes that play a role in responding to cellular stress by activating the double-stranded DNA repair process. The result inferred that mutations in these two genes cause DNA instability, making cells more susceptible to DNA-destroying agents such as Cisplatin and its derivative, Carboplatin [66], and PARP inhibitors. In addition, most BRCA mutations are associated with hereditary breast cancer, which is the most well-known cause of hereditary cancer predisposition [67,68].

The lifetime risk of breast cancer in carriers of BRCA1 and BRCA2 mutations is 45–80%. Characterized TNBC is more aggressive and has a higher tumor rate. About 80% of tumors have BRCA1 mutations. Despite the risk of a more aggressive tumor phenotype, most investigations have failed to show that BRCA mutation carriers have different clinical outcomes [69]. The cumulative risk of developing breast cancer at the age of 70 for carriers of BRCA mutations is 65% for BRCA1 and 45% for BRCA2. BRCA2-related breast tumors are dominantly ER-positive and p53 negative, while BRCA1-related breast tumors are more often in triple-negative breast cancer (TNBC) and p53 positive [70].

Platinum agents, such as anthracyclin and antimetabolite, are administered in the same metastatic setting and adjuvant as other conventional chemotherapy. In phase II clinical trials, platinum agent monotherapy was found to be effective in patients with BRCA1/2 mutations [66]. Furthermore, the advantage of Cisplatin in conjunction with Gemcitabine is applicable [68]. A clinical study using PARP inhibitors, such as Olaparib and BSI-201, is now ongoing and shows clinical efficacy in the treatment of BRCA1/2-related breast, ovarian, and prostate cancers, as well as sporadic basal-like breast cancers [71].

##### Poly-ADP Ribose-Polymerases (PARP)

The poly ADP-ribose polymerase (PARP) enzymes repairs DNA damage for maintaining BRCA-mutated cell viability in healthy cells and cancer. Several studies have reported that drugs that interfere with or inhibit the PARP enzyme make it more difficult for cancer cells with BRCA1/2 mutations to repair DNA damage. Cancer cells get a higher chance of survival as a response to this. On the other hand, PARP inhibitors make certain cancer cells less likely to survive DNA damage [72,73].

Clinical trials evaluating the oral PARP inhibitor olaparib in BRCA1/2-positive metastatic breast cancer are currently underway, with interim results showing efficacy [74]. Veliparib is another PARP inhibitor presently being assessed for metastatic TNBC combined with paclitaxel and Carboplatin [75]. Lynparza (Olaparib) and Talzenna (Talazoparib) have been PARP inhibitors that were approved to treat advanced HER2-negative breast cancer in people with BRCA1/2 mutations. Additionally, Atezolizumab, combined with Abraxane chemotherapy drug (chemical name: albumin-bound paclitaxel or nab-paclitaxel), is approved as the first treatment for advanced three-negative or metastatic local metastatic non-resection [73,75].

##### Histone Deacetylase (HDAC)

Histone acetyltransferases (HATs) catalyze the reversible process of lysine acetylation at the ε-amino group of proteinogenic lysine residues. Histone acetylation neutralizes the positive charge of lysine residues, correlated to chromatin relaxation and active gene transcription [76]. Besides, histone deacetylases (HDACs), which are functional antagonists of HATs, remove the acetyl groups [77], thus leading to a compressed chromatin structure (heterochromatin) and subsequently suppressing gene transcription [78]. TNBC agents that inhibit histone deacetylase (HDAC) play an important role in gene expression, cell proliferation, and survival [79,80]. 

Currently, Entinostat is an HDAC inhibitor that has been proven to have anti-CSC activity in TNBC stem cells. Entinostat treatment reportedly inhibited TNBC stem cell, tumor growth, and miR-181a expression in TNBC cell lines, as well as inhibiting lung metastases in an in vivo study [81]. Furthermore, in vivo and in vitro studies showed that combining entinostat, retinoic acid, and doxorubicin induced apoptosis and differentiation of TNBC stem cells [82].

An in vivo study showed that Panobinostat (LBH589) decreased cell survival and cell cycle development at the G2/M stage in TNBC cell lines. It also increased the acetylation of the histones H3 (Lys3) and H4 (Lys8) [79]. The drug panobinostat reversed the M phenotype in invasive breast carcinoma via inducing and upregulating cadherin-1 (CDH1) as a Wnt signaling component. In an in vivo investigation, the combination of salinomycin with panobinostat significantly inhibited the growth of TNBC stem cells in TNBC patient-derived xenografts. It inhibited cell cycle progression, regulated EMT, and increased apoptosis in TNBC stem cells in a synergistic manner [83].

##### p53

p53 is a known oncogene, the tumor suppressor gene. It is responsible for DNA damage repair, as well as apoptosis in cases of no replacement DNA damage or influencing, cell cycle arrest, necrosis, or autophagy. Mutations in p53, usually in TNBC, are approximately 60–70% [16].

#### 3.2.3. Targeting of Intracellular and Signaling Pathways

##### Androgen Receptor

Androgen receptors (AR) are hormonal steroid receptors that include nuclear receptor families and estrogen, glucocorticoid, mineralocorticoid receptors, and transcription factors. Characteristic of the androgen receptor, having overexpression involves a subtype of TNBC [84,85]. It links a transcription factor that controls specific genes, stimulates or suppresses cell proliferation and apoptosis, and activates signaling pathways [14,86,87]. Androgen receptor overexpression can be seen in 70–90% of breast cancers, with 10–50% of TNBC resulting from that expression [88,89].

Research on the relationship between AR and decreased relapse-free survival [90], higher mortality rate [91], or making survival benefit [92,93] are controversial. However, this class of TNBC has become a promising target for anti-androgen therapy. 

Bicalutamide is an AR inhibitor used in phase II trial studies in metastatic breast cancer patients [94]. Enzalutamide is an inhibitor of AR nuclear localization that has been well-tolerated in phase II clinical trials, with a CBR of 35% at 16 weeks and a median PFS of 14% [89,95,96]. In a phase II trial, seviteronel (INO-464), an oral selective cytochrome P450c17a (CYP17), 17,20-lyase (lyase), and androgen receptor (AR) inhibitor, showed promising antitumor activity in TNBC patients [97].

##### Heat Shock Protein 90 (HSP90)

Hsp90 expression levels were found in all subtypes of breast cancer receptors [98]. TNBC was sensitive to Hsp90 inhibition in preclinical and in vitro studies due to the downregulation of the Ras/Raf/MARK pathway [99]. Hsp90 interacts with estrogen receptors (ER), angiogenesis transcription factor HIF-1alpha, tumor suppressor p53 protein, antiapoptotic kinase Akt, Raf-1-MAP kinase, and a family of receptor tyrosine kinases including HER2 [100].

The HSP90 inhibitor (17-DMAG) is more sensitive in the LAR class of TNBC cell lines than basal-like or mesenchymal cell lines [101]. In a phase II clinical trial, single-agent ganetespib was shown to have good tolerability and be able to decrease lung tumor metastases in TNBC patients [102]. Since the clinical study of the combination of onalespib and talazoparib (PARPi) has been withdrawn, the clinical study was conducted using a combination of onalespib and paclitaxel instead [54]. According to in vivo and in vitro experiments, simvastatin acts as an Hsp90 inhibitor in TNBC cells by inhibiting the development of the K292acetylated Hsp90/Cdc37 complex. Simvastatin with Panobinostat (LBH589) is a deacetylase inhibitor based on hydroxamic acid that targets TNBC specifically [103].

##### Cyclin-Dependent Kinases (CDKs)

CDKs are the only cell cycle and factor transcriptional regulators. Overexpression of CDKs, such as CDK4 and CDK6, is a common characteristic of many cancers, including TNBC. Most of the inhibitors of CDKs have exhibited anti-TNBC activity in vivo and in vitro. Dinaciclib was shown to be a pan-CDK inhibitor in a phase I clinical trial, with no toxicity issues in combination with epirubicin (dinaciclib 20 mg/m^2^ on day one and epirubicin 75 mg/m^2^ on day 2 of a 3-week cycle) in 9 TNBC patients [104]. The Dinaciclib combination with pembrolizumab is being studied in phase I and phase II clinical trials with a dose of 33 mg/m^2^ on cyclin D1 in 8 days from a 21-day cycle [105]. However, phase II clinical studies are now investigating Trilaciclib; a CDK4/6 inhibitor, ribociclib; a CDK6 inhibitor, cyclin D1/CDK4, and PF-06873600, abemaciclib, CDK2/4 inhibitors.

##### Phosphoinositide 3-Kinase (PI3K)/AKT/Mammalian Target of Rapamycin (mTOR) Pathway

PI3K is a signal transducer that reduces activated receptor tyrosine kinases (RTKs). The signaling pathway of PI3K is in association with AKT and mTOR, known as the PI3K/Akt/mTOR pathway [106]. Activating this pathway in TNBC has a 10–21% impact on cell cycle regulation, cell proliferation, and quiescence [107]. Activated mTOR is also involved in the metabolism and migration of cells. PI3K, AKT, and mTOR inhibitors were used to inhibit this pathway. PI3K inhibitors, which are taselisib, gedatolisib, BKM120, BYL719, AZD8186, BEZ235, CUDC-907, GDC-0941, and PQR309, have been used in phase I clinical trials for TNBC. AKT inhibitors including AZD5363, ONC201, ARQ 092, ritonavir, and GSK2141795 are also in phase I or II clinical trials [46,108,109].

##### RAF-MEK-ERK Pathway

The higher expression of various genes in the Raf/MEK/ERK pathway and AKT/MEK pathway [110] was involved in the TNBC subtype. It is important to target this signaling pathway in TNBC. Trametinib, a MEK1/2 inhibitor, showed more upregulation and activation of receptor tyrosine kinase [111]. A clinical trial in 50 TNBC patients found that either a single medication or a combination of drugs with an AKT inhibitor (GSK2141795) had low effectiveness. Trametinib, in conjunction with spartalizumab (anti-PD1), was the subject of another clinical trial [112]. Another MEK inhibitor, in combination with BKM120 and BEZ235, completed a clinical trial, but the findings were not published.

##### Janus Kinase (JAK)

The JAK-STAT signaling pathway in mammalians consists of four Janus kinase domain-containing proteins, JAK1, JAK2, JAK3, and tyrosine kinase 2 (TYK2), as well as seven signal transducers and activators of transcription—STATs (STAT1, STAT2, STAT3, STAT4, STAT5A, STAT5B, and STAT6) [113]. Deregulation of this pathway in oncogenic phenotypes involved tumorigenesis, proliferation, angiogenesis, oncogenic, survival, anti-apoptosis, and immune response [114]. 

The Janus kinase 2 (JAK-2) gene was located on chromosome 9p24.1. Its protein is a tyrosine kinase by the JAK-STAT pathway, which shows that TNBC tumors are related to a poorer prognosis and shorter survival [115,116], and amplified JAK2 are more sensitive to the effects of specific inhibitors in TNBC cells [114]. 

Cell proliferation in the mammary gland develops during puberty and pregnancy, and cancer is all mediated by the JAK-STAT pathway. Ruxolitinib is a tyrosine kinase inhibitor that targets JAK1 and JAK2. This drug would affect the importance of JAK2 in TNBC. These include combination with pembrolizumab in advanced TNBC patients, paclitaxel, doxorubicin or cyclophosphamide, and paclitaxel to treat triple-negative inflammatory breast cancer. Ruxolitinib did not meet the primary efficacy target as a single agent in this refractory patient population, despite the evidence of on-target activity [117].

##### Signal Transducer and Activator of Transcription 3 (STAT-3)

STAT3 was discovered binding to DNA in response to interleukin-6 (IL-6) and epidermal growth factor (EGF) during inflammation [118,119]. Overexpressed signal transducer and activator of transcription 3 (STAT3) are highly associated with cancer initiation, metastasis, cell survival, cell cycle progression [66,119], proliferation, migration, invasion, anti-apoptosis, angiogenesis, chemoresistance, immunosuppression, and stem cell self-renewal and differentiation of TNBC cells of clinical and preclinical studies [120,121]. STAT3 inhibitors have since been shown to be effective in inhibiting TNBC tumor growth and metastasis in clinical trials.

Currently, STAT3 small molecule inhibitors and targeting strategies have shown anti-cancer activity in TNBC in vivo and in vitro [120,122]. STAT3 minor molecule inhibitors, which are more selective and efficacious, are critical for TNBC prevention and therapy [123].

TTI-101 and OPB-51602 are small molecules that inhibit STAT3 activation via inhibiting JAK-mediated tyrosine phosphorylation. These molecules connect to the phosphotyrosine peptide binding site inside of the Src homology 2 molecules (SH2). A phase I study of TTI-101 and OPB-51602 is currently recruiting breast cancer patients. The STAT3 inhibitor AZD9150 is used to treat patients with advanced solid tumors in phase I and II clinical studies, either alone or in combination with chemotherapy [124].

##### Wnt/β-Catenin Signaling Pathway

TNBC can be expressed by Wnt signaling. Wnt signaling acts as a complex antagonist of β-catenin destruction to affect cancer cells and metastases and control the immune system. Research by De et al. (2016) shows that TNBC cells migrate and become invasive clonogenic through upregulation of the Wnt/β-catenin pathway. Wnt/β-catenin plays an essential role as a regulator adhesion cell. The research was focused on the role of β-catenin as a therapeutic agent in TNBC.

The canonical Wnt pathway is a transcription coactivator on TCF/LEF that induces the accumulation of β-Catenin protein and its translocation from the cytoplasm into the nucleus, stimulating the expression of numerous genes involved in cell proliferation, cell migration, and so on. The study shows that increasing regulation and maintaining Wnt/β-Catenin signaling in TNBC is associated with metastasis and poor prognosis. 

LGK-974, a Porcupine inhibitor, is a small molecule that inhibits the Wnt signaling pathway in vitro and in vivo by decreasing LRP6 phosphorylation and Axin2 expression. A single drug has been tested in phase I clinical studies in people with TNBC [125]. In vitro studies have revealed that combining LGK-974 with a PI3K/Akt/mTOR inhibitor reduces cell viability and enhances anti-cancer efficacy in TNBC cell lines [126,127].

In vitro and in vivo studies demonstrate that CWP232228 inhibits the stem cell growth in TNBC cell lines by antagonizing the binding of β-catenin to T-cell factor (TCF) in the nucleus, which is required for breast cancer metastasis and recurrence [128]. PRI-724 is a CRB protein inhibitor [125]. OMP-18R5 (Vantictumab) is a monoclonal antibody that binds to Frizzled7 in the extracellular domain and suppresses the development of human tumors in a xenograft model while having a synergistic effect with chemotherapeutic agents [129]. OTSA101 is an inhibitor of frizzled10, and OMP-54F28 (Ipafricept) is a fusion protein cysteine-rich domain of frizzled-8 receptors with the immunoglobulin for competition in ligands as antagonist Wnt signaling [130,131].

#### 3.2.4. Targeting of Cell Surface

##### Vascular Endothelial Growth Factor Receptor 2 (VEGFR2)

The vascular endothelial growth factor receptor (VEGFR2) is a receptor tyrosine kinase that regulates angiogenesis and pathogenesis in breast cancer [132]. VEGF, the ligand to VEGFR2, impacts ligand expression involving tumor invasion and metastasis in TNBC [133,134,135,136]. Patients who have had TNBC surgery have significantly higher levels of VEGF and shorter survival [137].

VEGFR inhibitors, such as bevacizumab, ramucirumab, VEGFR receptor blockers, receptor mimetics (such as aflibercept), and sorafenib, are small-molecule tyrosine kinase inhibitors [134]. Patients with TNBC who were treated with the medication of sunitinib for metastasis alone had a worse prognosis than those in a phase II trial [133].

##### Epidermal Growth Factor Receptor (EGFR)

The epidermal growth factor receptor (EGFR) is an HER family tyrosine kinase receptor that is found in a variety of epithelial tumors [138]. EGFR activation has an essential function in the survival of many solid tumors, including metastasis, cell proliferation, invasion, cell cycle progression, differentiation, angiogenesis, and apoptosis. Overexpression EGFR in breast cancer cells is approximately 15–45% [139], and about 50% in TNBC [140], which is negatively correlated with patient survival rates [141]. Anti-EGFR monoclonal antibodies such as cetuximab (SCT200), and EGFR small-molecule tyrosine kinase inhibitors such as gefitinib and erlotinib, are used to block the EGFR signaling pathway in TNBC [138]. Afatinib has been included in clinical studies, however, its status is still unclear. In a phase II trial, erlotinib, in combination with paclitaxel nanoparticle formulation and bevacizumab, showed excellent tolerability [142]. 

##### Fibroblast Growth Factor Receptor (FGFR)

FGFR2 is overexpressed in TNBC cells by around 4%, while FGFR1 and FGFR2 mutations were found in roughly 16% and 13% of TNBC patients, respectively [143]. The expression of FGFR2 in TNBC patients is an independent prognostic factor. Approximately 4% of TNBC have amplification of the FGFR2 gene on chromosome 10q26. Nevertheless, it appears to be a rare occurrence in other tumor subtypes, with just 1–2% of all breast cancers expressing it [144,145]. IM-412 is a small molecule tyrosine kinase inhibitor or monoclonal antibody in the TNBC subtype [146]. 

##### Trophoblast Antigen 2 (Trop-2) Inhibitor

Trop-2 is a cell surface receptor and an epithelial glycoprotein-1. Overexpression of Trop-2 can promote cancer cell proliferation, EMT, migration, invasion, and metastasis in a variety of epithelial malignancies. For example, Trop-2 was discovered in TNBC cells, with more than 85% of its expression in tumors [147,148]. 

Sacituzumab-bound tumor cells are killed by intracellular uptake and extracellular release of SN-38 [149]. Sacituzumabgovitecan-hziy (or IMMU-132, Immunomedics, or hRS7-SN-38) is a monoclonal antibody-drug combination in which SN-38, an active metabolite of irinotecan, is linked to the humanized antitrophoblast cell-surface antigen 2 (Trop-2) monoclonal antibody hRS7 IgG1 through the cleavable CL2 linker of TNBC patients in phase I/II clinical trial [150]. 

##### Glycoprotein Non-Metastatic B (GPNMB)

GPNMB is a type I transmembrane glycoprotein that is overexpressed in 40–60% of breast cancer cases, including triple-negative cases [151]. In phase I/II trials, glembatumumabvedotin (CDX-101), an antibody targeting GPNMB, exhibited a favorable safety profile on 42 patients with metastatic breast cancer [152,153]. However, the results of a further phase II clinical trial in TNBC patients with metastatic CDX-011 impact have yet to be reported.

## 4. Natural Compounds for TNBC Treatments

Natural compounds have the potential to be used as therapeutic agents in the treatment of TNBC. Some natural compounds and potential molecular targets in the TNBC signaling pathway have been identified as an anti-cancer treatment. It has recently been shown that determining the concentration or dose of biological substances with comparable chemical components and effects, particularly for various anti-cancer treatment medicines, does not necessarily result in the same anti-cancer effectiveness [154]. As a result, the concentration/dose of natural components in anti-cancer treatment should be considered.

Many natural substances with anti-cancer activities have gotten a lot of attention due to their various behaviors. The conventional treatment approach to breast cancer appears to be limited by several problems. The most critical problem is the toxic effects of treatment resistance. As a result, these numerous cancer treatments have been developed, many of which involve natural compounds such as vinca alkaloids, taxanes, podophyllotoxins, and anthracyclines (doxorubicin) [155]. Plant-derived compounds have a promising synergistic relationship with a variety of chemotherapy regimens, enhancing their effectiveness. Genistein and doxorubicin have a synergistic effect and boost the tamoxifen effect, and pomegranate, which promotes the tamoxifen-induced cell viability inhibition, are two examples of these combinations. Natural materials are also preferred over conventional therapies since they are easily accessible in the natural environment and typically have fewer side effects on healthy human cells.

**Figure 3:**
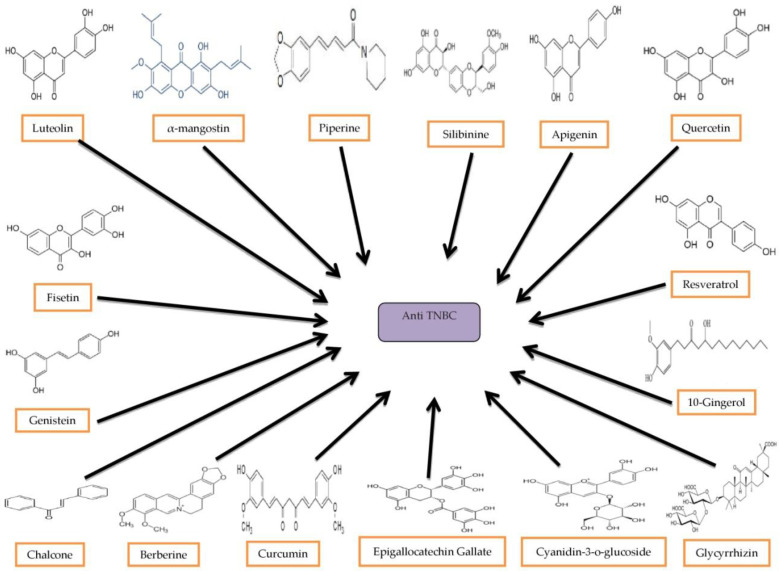
Bioactive compounds from different sources have shown anti-TNBC.

Many plant-derived natural compounds have anti-cancer properties, including quercetin, formononetin, calycosin, polyphenols, bioflavonoids, carotene, vitamins, and minerals [156,157,158]. They can suppress cell growth, migration, and metastasis by targeting irregular/irregular signaling pathways present in TNBC, such as Wnt/β-Catenin, NF-κB, PI3K/Akt/m-TOR, PD-1/PD-L1, LAG-3, CTLA-4, STAT-3, EGFR, Trop-2, RAF/MEK/ERK, JAK, Glycoprotein NMB (GpNMB), and hedgehog pathways. Here, we highlight the potential of using phytochemicals (luteolin, α-mangostin, piperine, silibinine, apigenin, quercetin, fisetin, resveratrol, genistein, 10-gingerol, chalcones, berberine, curcumin, epigallocatechin gallate, cyanidin-3-o-glucoside, and glycyrrhizin) in the treatment of TNBCs and their mechanisms of action. These natural compounds were collected after a thorough search of reports and studies on the Internet and in databases. Bioactive compounds from different sources in various plants are shown in Figure 3.

### 4.1. Luteolin

Luteolin is a flavonoid compound found in many plants such as carrots, celery, broccoli, perilla leaf, and seed [159]. A study that used two methods to determine the mechanisms of luteolin on TNBC metastasis (in vitro with a xenograft model and in vivo with MDA-MB-231 and BT5-49 cell lines), found that Luteolin dose-dependently inhibited cell migration and invasion, reversed epithelial–mesenchymal transition (EMT), and suppressed the expression of β-catenin mRNA that then suppressed metastases to the lung of breast cancer cells at a concentration of 100 μM. The result indicated that luteolin had a potent therapeutic effect on invasion and metastasis of TNBC, which may be involved in the reversal of EMT by down-regulation of β-catenin [160]. The other research showed in vivo studies of luteolin suppressed lung metastasis of TNBC in MDA-MB-231 (4175) and MDA-MB-435 cell lines LM2 with concentrations of 40 mg/kg and 20 mg/kg, respectively. Luteolin significantly inhibited tumor cell migration to reduce VEGF levels and block VEGF receptors, with IC_50_ of 10 µM in vitro and in vivo [161]. In addition, luteolin from *Taraxacum officinale* extract can inhibit Nrf2 in breast cancer stemness (Cripto1, CD44, ALDH1, ABCG2, NANOG, OCT4, and Sirt3) and chemoresistance, with IC_50_ value 1 µM in an MDA-MB-231 cell line in vitro study [162].

### 4.2. α-Mangostin

α-mangostin is isolated from *Garcinia mangostana* Linn with the mechanism of action as anti-proliferation, apoptosis, suppressed angiogenesis, and metastases [163]. According to a study in 2018, α-mangostin could significantly reduce the development of the spheroids in an MDA-MB-231 cell line, with an IC_50_ value of 1.25 µg/mL. This finding points to a novel anti-cancer property of α-mangostin that could be used to improve conventional drug penetration into tumor bulk [164]. Another research reported that α-mangostin suppressed the proliferation, migration, and invasion of the PI3K/Akt signaling pathway by targeting RXRα and cyclin D1 in vitro and in silico studies. This compound was inhibited in the MDA-MB-231 cell line, with an IC_50_ value of 11.37 µM [165].

### 4.3. Piperine

Piperine is an alkaloid found in the fruits of black pepper (*Piper ningrum* Linn.) and long pepper (*Piper longum* Linn.) [166]. The research on dose-dependent reduction in the number of TNBC cells (MDA-MB-468, MDA-MB-231) and estrogen receptor-expressing breast cancer cells (MCF-7, T-47D) discovered that piperine decreased the percentage of TNBC cells in the G2 phase of the cell cycle and inhibited the in vitro growth of p53-deficient. Piperine also inhibited TNBC cell migration and expression of matrix metalloproteinase-2 and -9 mRNA in vitro and in immune-deficient mice in vivo with the IC_50_ value of 50 µM [167].

### 4.4. Silibinin

Silibinin is a major bioactive flavanone. It has biological activity in a variety of cancer models such as breast and lung cancers by inhibiting cell proliferation, invasion, and angiogenesis. In the research using Hs578T, MDA-MB-231, BT474, T47D, HCC1806, and HCC1143 cell lines, silibinin significantly decreased TGF-β2-induced FN, MMP-2, and MMP-9 expression levels and suppressed the lung metastasis of TNBC cells. It also decreased TGF-β2 mRNA expression levels but not that of TGF-β1 in TNBC cells, and cell migration, as well as basal fibronectin and MMP-2 expression levels, decreased as well in response to silibinin in vitro and in vivo studies with the IC_50_ value of 50 µM [166]. Another study reported that silibinin inhibited the gene-specific transcriptional activation of MMP-2 expression and suppressed the phosphorylation of the Jak2/STAT3 signaling pathway by blocking the STAT3 nuclear translocation and DNA-binding activity, resulting in reduced cell migration and invasion with the IC_50_ value of 200 µM in MDA-MB-231 cell line [168].

### 4.5. Apigenin

Apigenin is a natural flavonoid compound and has an effect on diabetes, amnesia and Alzheimer’s disease, depression, insomnia, and cancer [169]. Apigenin is found to be able to decrease the expression of target genes, such as CTGF and CYR61 and YAP/TAZ activity in TNBC cells and disrupt the YAP/TAZ-TEADs protein–protein interaction in MDA-MB-436 cells. Meanwhile, in MDA-MB-231 cells, apigenin disrupts the TAZ–TEADs interaction but has no evidence of the interaction between YAP and TEADs, with the IC_50_ value of 20 µM [132]. In addition, apigenin can inhibit pro-inflammatory proteins such as CCL2, TNF-α, and IL-6 at extremely high concentrations in MDA-MB-468 compared to MDA-MB-231 cell lines, with an IC_50_ value of 40 µM [170].

### 4.6. Quercetin 

Quercetin is a plant-derived flavonoid found in fruits, vegetables, and tea, which is known to have multiple biological actions such as antioxidant, anti-inflammatory, and anti-cancer. Quercetin induces apoptosis and cell cycle arrests by modulation of Foxo3a activity and inhibition of JNK activity that reduced the signaling activities of p53, p21, and GADD45 in the MDA-MB-231 cell line, with the IC_50_ value of 20 µM [171]. Other research shows that quercetin significantly inhibits nuclear accumulation of β-catenin with reduced target genes such as cyclin D1 and c-Myc by inducing the E-chaderin expression and the ability to modulate a mesenchymal-to-epithelial transition (MET) in MDA-MB-231 and MDA-MB-468 cell lines. The in vitro study had an IC_50_ value of 50 µM [172]. Additionally, the in vivo study showed that quercetin inhibited tumor growth and FASN expression in tumor xenograft with a concentration of 50 mg/kg and induced apoptosis through down-regulation of caspase-3 activity, FASN, β-catenin, and Bcl-2 protein expression in the in vitro study. The IC_50_ values were 3 µM and 4 µM in MDA-MB-231 and MDA-MB-157 of TNBC cell lines, respectively [173].

### 4.7. Fisetin

Fisetin is one of the major flavonoids from many fruits and vegetables such as strawberries, apples, persimmons, grapes, onions, and cucumbers [174]. Fisetin dose-dependently inhibits cell proliferation, migration, and invasion in MDA-MB-231 and BT549 cells. In vitro assay demonstrated that fisetin suppressed phosphoinositol 3-kinase (PI3K)/Akt/GSK-3β signaling pathway but upregulated the expression of PTEN mRNA and protein in a concentration-dependent manner. On the other hand, in vivo tests, with a concentration of 100 mg/kg, indicated that fisetin could inhibit the growth of primary breast tumors and reduce lung metastasis while increasing the expression of EMT molecules and PTEN/Akt/GSK-3β with an IC_50_ value of 100 µM [175].

### 4.8. Resveratrol

Resveratrol is a non-flavonoid polyphenolic compound from wine and grape juice, also synthesized in grape leaves and grape skins. It is reported that resveratrol promoted the apoptosis of TNBC cells by reducing POLD1 expression, thereby activating the respective apoptosis pathways in the MDA-MB-231 cell line by in vitro and in vivo assays having an IC_50_ value of 50 µM [176]. Another research reported that resveratrol at an IC_50_ value of 185 µM combined with 14 µM cisplatin inhibited fibronectin, vimentin, PI3K/Akt, Smad2, Smad3 JNK, ERK, Nf-_K_B expressions by TGF-β1, and increasing E-cadherin expression. This compound can also inhibit migration, invasion, and tumor growth within in vitro and in vivo studies in MDA-MB-231 [177].

### 4.9. Genistein

Genistein (Gen) is a natural isoflavone with biological activities such as anti-breast cancer [178]. In a dose-dependent manner, genistein induced apoptosis and cell cycle arrest in the G2/M phase. Gen inhibited NF-κB activity by the Nocth-1 signaling pathway, as well as downregulated cyclin B1, Bcl-2, and Bcl-xL expression in the MDA-MB-231 cell line at an IC_50_ value of 20 µM. Further preclinical and clinical studies are warranted to investigate the application of Gen for the treatment of TNBC [179]. Other research showed that Gen inhibited CDK1 kinase activity by phosphorylation on the Thr14 and Tyr15 sites by inducing G2/M cell cycle arrest, apoptosis, and DNA damage response pathways such as ATR and BRCA1 activation. An IC_50_ value of 40 µM was present in the MDA-MB-231 cell line [180].

### 4.10. (10)-Gingerol

(10)-gingerol is found in ginger (*Zingiber officinale* Roscoe) oleoresin from a fresh rhizome. The results reported that (10)-gingerol induced metastatic dissemination, including lung, bone, brain, and apoptosis death in mouse and human TNBC (MDA-MB-231) cell lines in vitro and in vivo. It also inhibited 4T1Br4 orthotopic tumor growth, with a concentration of 10 mg/kg in the in vivo study. Furthermore, the in vitro study obtained the IC_50_ value of 100 µM in the MDA-MB-231 cell line [181]. The other research showed inhibited mitogen-induced activation of Akt and p38MAPK and the suppressing of epidermal growth factor receptor expression. The result reported cell migration and invasion through the suppression of MMP-2 activity, with an IC_50_ value of 10 µM in the MDA-MB-231 cell line by in vitro study [182].

### 4.11. Chalcones

Chalcones is a natural flavonoid from many flowers and plants, including fruits and vegetables [183,184]. It has pharmacological activities such as hypertension, infectious diseases, neurological disorders, and cancer [185]. Chalcone, extracted from Cardamonin, induces invasive, migration, and reverses epithelial–mesenchymal transition (EMT) by downregulation of Wnt/β-catenin signaling in BT-549 and MDA-MB-231 cell lines. This result significantly inhibits the phosphorylation of GSK3-β by inhibiting Akt activity. The in vitro study and concentration of 5 mg/kg in vivo study had an IC_50_ value of 20 µM in BT-549 and MDA-MB-231 cell lines [186].

### 4.12. Berberine

Berberine is a natural isoquinoline alkaloid compound isolated from the stems and roots of plants such as Berberis vulgaris, Berberis asiatica, Berberis aristata, Coptidis japonica, Coptidis japonica, Coptidis rhizome, Coptidis chinensis, Mahoniaaqui folium, and Mahonia beale [187,188]. Berberine significantly induced apoptosis and had the most sensitive reaction to HCC70, BT-20, and MDA-MB-468 cell lines, with IC_50_ values of 0.19 µM, 0.23 µM, and 0.48 µM, respectively. Berberin also induced cell cycle arrest at G1 and/or G2/M phases in MDA-MB-468 and HCC70 cell lines and S phase in BT-20 cell line. Berberine induced apoptosis with an IC_50_ value of 1 µM in all of the cell lines by in vitro study. The research suggests berberine as a potential candidate for TNBC therapy [189].

### 4.13. Curcumin

Curcumin induces apoptosis and decreased expression levels of extracellular regulated protein kinase (ERK1/2), pERK1/2, EGFR, and pEGFR in MDA-MB-231 cells [190]. The research suggested curcumin as a potential anti-TNBC due to its ability to promote apoptosis, and to block the cell cycle of TNBC cells (MDA-MB-231) by inhibiting restoring DLC1 and EZH2 expression; it also inhibited the migration, invasion, and proliferation in vitro and in vivo studies with an IC_50_ value at 40 µM for both MDA-MB-231 and MDA-MB-468 cell lines [191]. Other research showed that curcumin inhibited the SIK3-mediated cyclin D upregulation in the G1/S cell cycle and inhibited cell growth during epithelial-mesenchymal transition (EMT), with an IC_50_ value of 25 µM in the MDA-MB-231 cell line by in vitro and in vivo studies [192].

### 4.14. Epigallocatechin Gallate

Epigallocatechin gallate (EGCG) is a major natural component of green tea. EGCG has been evaluated in some clinical trials. It has been reported that Epigallocatechin gallate suppressed the growth, migration, and invasion of TNBC cells by inhibiting VEGF gene expression in the Hs578T cell line [193]. Wnt/β-catenin activation was downregulated by EGCG. However, upregulation of Wnt/β-catenin extinguished the inhibitory effects of EGCG on lung cancer [194]. Wnt/β-catenin signaling was suppressed by EGCG by promoting GSK-3β and PP2A-independent phosphorylation/degradation of β-catenin with the IC_50_ value of 80 µM [195]. Hong et al. (2017) reported that EGCG can also inhibit the β-catenin pathway, phosphorylation of Akt, and cyclin D1 expression, with an IC_50_ value of 200 µM in the MDA-MB-231 cell line [196]. Other research showed that the synthesis of EGCG analogues are diesters (G28, G37, and G56) and monoesters (M1 and M2) inhibiting the lipogenic enzyme fatty acid synthase (FASN) with an IC_50_ value of 1.5 µM in the MDA-MB-231 cell line [197].

### 4.15. Cyanidin-3-o-Glucoside

Cyanidin-3-o-glucoside is an anthocyanin from the flavonoids group. Cyanidin-3-o-glucoside was reported to effectively promote apoptotic cell death in MDA-MB-231, MDA-MB-436, and BT20 cell lines by inhibiting the estrogen receptor alpha 36 (ERα36) and EGFR/Akt signaling with an IC_50_ value of 500 µM [198]. Cyanidin-3-o-glucoside also downregulates β-catenin and methylguanine-DNA methyltransferase (MGMT). In addition, miR-214-5p mimics β-catenin and downregulates MGMT in LN-18/TR cells, whereas miR-214-5p inhibitors have the opposite effect; miR-214-5p inhibitors significantly block Cyanidin-3-o-glucoside-induced downregulation of β-catenin and MGMT [199]. 

### 4.16. Glycyrrhizin

Glycyrrhizin is a natural compound from licorice root and its metabolite, glycyrrhetinic acidis, is potent against TNBC by inhibiting cell proliferation. Glycyrrhetinic acid exhibits a synergistic effect of etoposide and upregulation of TOPO 2A with an IC_50_ value of 20 μM in MDA-MB [200]. The other research showed that glycyrrhizic acid from licorice root extracts inhibited intracellular and reactive oxygen species—mitochondrial, cell death, and autophagy by the nuclear translocation of apoptosis-inducing factors (AIF) and LC-3 in the MDA-MB-231 cell line, with an IC_50_ value of 20 µM in vitro study [201].

Some of the natural compound’s activities and its mechanism are summarized in Table 2.

### 4.17. Ilamycin E

Ilamycin E from marine actinomycete isolated from deep sea-derived *Streptomyces atratus*, has anti-TNBC activities with inhibited G1/S cell cycle progression and induced apoptosis by activation of endoplasmic reticulum (ER) stress, increasing the expression of CHOP and suppressing Bcl-2 transcriptionin cell lines HCC1937 and MDA-MB-468 of TNBC, with IC_50_ values of 14.24 μM in HCC1927 and 24.56 μM in MDA-MB-468, with IC_50_ values of 14.24 μM in HCC1927 and 24.56 μM in MDA-MB-468 cell lines [202].

### 4.18. Schisandrin A

Schisandrin A, a bioactive phytochemical, is one of the representative lignans species from the fruit of *Schisandra chinensis* Turcz. (Baill.). It has biological activity such as anti-inflammation and anti-oxidative stress [223]. A study found that Schisandrin A suppressed the development of TNBC cells in vitro and in xenograft mouse models on MDA-MB-231 and BT-549 cells by inducing cell cycle arrest and cell death, as well as an overactivation of Wnt signaling in TNBC cells. The IC_50_ values against MDA-MB-231 and BT-549 cells are 1.45 μM and 6.85 μM, respectively [203]. 

### 4.19. Ampelopsin E 

Ampelopsin E, an oligostilbene derived from the Dryobalanops species, has anti-cancer and anti-inflammatory activities. It reduces invadopodia formation, migration, transmigration, and invasion of MDA-MB-231 cells by decreasing the expression of PDGF, MMP2, MMP9, and MMP14 significantly (*p* < 0.05). The percentage of cell viability of Ampelopsin E is higher than 80% at a concentration of 15 μM [204]. 

### 4.20. Aurantoside C

Aurantoside C (C828), isolated from Sponge (*Manihinealyn beazleyae*), inhibited the phosphorylation of Akt/m-TOR and NF-kB pathways and increased the phosphorylation of p38 MAPK and SAPK/JNK pathways, leading to apoptosis in TNBC cells. C828 was effective in reducing cell viability in SUM159PT, MDA-MB-231, and SUM149 with the IC_50_ values of 0.01 μM, 0.01 μM, and 0.02 μM, respectively, compared to non-TNBC cells and chemotherapeutic drugs (doxorubicin and cisplatin) on SUM159PT cells after 24 h of treatment [205].

### 4.21. Amyris texana 

The discovery of isoxazole compound (CIDD-0067106) from *Amyris texana* inhibited the phosphorylation of Akt/mTOR and NF-κB signaling pathways, a model of the Luminal Androgen Receptor (LAR). The result showed IC_50_ of 0.8 μM in MDA-MB-453 cells [206].

### 4.22. Sequesterpenoid (Tussilago farfara)

Sequesterpenoid was isolated from Farfarae Flos (*Tussilago farfara*). The sequesterpenoid fraction used countercurrent chromatography (CCC) and isolation, using preparative-HPLC. This compound showed inhibited JAK–STAT3 signaling pathway and suppressed the expression of STAT3 target genes, inducing apoptosis of TNBC MDA-MB-231 cells by extrinsic and intrinsic pathways in the in vitro and in vivo studies. The result of the IC_50_ values is 0.18 μM compared to the positive control of *Staurosporine* [207].

### 4.23. Diterpen Jatrophone

Diterpen Jatrophone is derived from the plant *Jatropha isabelli*. *Jatrophone* isolated was purified by normal-phase silica gel column chromatography. This study compared various TNBC subtypes of MSL-TNBC cell lines in MDA-MB-231 versus MDA-MB-157 with BL-1 subtype TNBC cell lines in HCC-38 versus MDA-MB-468. This compound showed the capability to inhibit the proliferation of the oncogenic WNT10B/β-Catenin/HMGA-2 signaling axis. However, the IC_50_ values were 2 μM in MDA-MB-231 and 3.5 μM in MDA-MB-157 cell lines, whereas in HCC38 and MDA-MB-468 cell lines were 2 μM and 1 μM, respectively [208,209].

### 4.24. Naringin/Flavonoid

Naringin is a flavonoid compound specifically of the flavanone subgroup. This compound of purity ≥95% uses HPLC. Naringin can induce G1 cell cycle arrest, inhibit cell proliferation, and promote cell apoptosis by regulating p21, survivin, and suppressed β-catenin signaling pathway with IC_50_ values of 200 μM in MDA-MB-231, MDA-MB-468, and BT-549 cell lines [210].

### 4.25. Myrothamnus flabelli folius

*Galloylquinic acids* from *Myrothamnus flabella* *folius* extracts have the potential an anti-cancer. They inhibit the growth of TNBC cells with a concentration of 31.125 μg/mL in BT-549 and MDA-MB-231 cell lines [211].

### 4.26. Cryptotanshinone

Cryptotanshinone is a bioactive component from the dried roots of *Salvia miltiorrhiza* Bunge (Danshen) that is purified by normal-phase silica gel column chromatography followed by preparative TLC [224]. KYZ3 inhibited TNBC cell metastasis by decreasing the levels of MMP-9 which were directly regulated by activated STAT3. A STAT3 plasmid transfecting assay suggested that KYZ3-induced tumor cell apoptosis target STAT3 MDA-MB-231 and MDA-MB-468 cells by suppressing the growth of tumors resulting from subcutaneous implantation of MDA-MB-231 cells in vivo with IC_50_ values of 0.68 μM and 0.86 μM in MDA-MB-468 [212].

### 4.27. Curcuma longa

Curcumin from rhizomes of *Curcuma longa* (C1386, purity >65%) was purified by column chromatography on silica gel using CHCl_3_/hexane 90:10 as eluent using TLC for monitoring the reaction. The result showed that analog curcumin (1–3) compounds can decrease the activity of the NF-κB transcriptional factor. The compounds inhibited TNBC cell lines with IC_50_ values of 1.30, 1.59, and 0.88 μM in the SUM149 cell and 0.41, 0.00, and 0.85 in MDA-MB-231, respectively, compared to curcumin [213].

### 4.28. Ganoderma lucidum

*Ganoderma lucidum* is a medicinal mushroom with anti-cancer activity. It was found to reduce cell adhesion, proliferation, survival, invasion, and downregulation of the STAT3 pathway. *Ganoderma lucidum* decreases the STAT3 pathway and the expression of OCT4, NANOG, and SOX2 in vitro, as well as in vivo on injected limiting dilutions (CD44+/CD24–) tumor models with IC_50_ values of 0.50 mg/mL in SUM-149 and 0.96 mg/mL in MDA-MB-231 cells [214].

### 4.29. Astragalus membranaceus

*Astragalus membranaceus* major components are comprised of polysaccharides, flavonoids, and saponins with a purity of 98%. It has pharmacology activities, such as immunomodulating, anti-oxidant, and anti-inflammatory [225]. The in vitro study reported that *Astragalus polysaccharides* inhibited the proliferation, invasion, and apoptosis of cell lines by the PIK3CG/AKT/BCL2 pathway, with an IC_50_ value of 2 mg/mL in MDA-MB-231 [216].

### 4.30. Vanicoside B

Vanicoside B, isolated from *Persicaria dissitiflora*, has been reported as an antiproliferative agent in cancer cells. Vanicoside B suppressed CDK8-mediated signaling pathways and the expression of epithelial−mesenchymal transition proteins and induced cell cycle arrest and apoptosis in MDA-MB-231 and HCC38 TNBC cells in vitro and in vivo study, with the IC_50_ values of 9.0 μM [217].

### 4.31. Eupalinolide J

Eupalinolide J is a new sesquiterpene lactone isolated from *Eupatorium lindleyanum* DC. It has various biological activities, including anti-inflammatory [226], anti-cancer [227], and anti-oxidant activities [228]. The purity of Eupalinolide J was above 95%. Eupalinolide J suppressed tumor growth by STAT3 signaling pathways in vitro and in vivo in the mouse xenograft model which induces apoptosis, mitochondrial membrane potential (MMP) disruption, proliferation, and cell cycle arrest at the G2/M phase. The IC_50_ values were 0.58 in MDA-MB-231 and 0.39 μM in MDA-MB-468 cells [218].

### 4.32. Chantaridin

Chantaridin is a terpenoid compound from the blister beetle *Mylabris phalerata* (Pallas). Chantaridin inhibited cell proliferation by inducing apoptosis and inhibiting autophagy, additionally leading to the conversion of LC3-I to LC3-II with suppressed Beclin-1 expression in vitro using flow cytometry and in vivo using nude mice of tumor xenograft with a dose of 10 mg/kg. The IC_50_ value is 5 µg/mL in MDA-MB-231 and MDA-MB-468 TNBC cell lines [219,229].

### 4.33. Cucurbitacin E

Cucurbitacin E was isolated from *Hemsleya delavayi* var. yalungensis (Cucurbitaceae). This compound was extracted with methanol followed by purification using silica gel column chromatography by monitoring TLC and spectroscopic. Cucurbitacin E has been reported to significantly decrease cell viability by inducing cell cycle G2/M phase arrest, decreased expression of cyclin D1, survivin, XIAP, Bcl-2, and Mcl-1 and increased activation of JNK, as well as inhibited AKT and ERK activation. The reported IC_50_ value is 0.2 μM in MDA-MB-468 and SW527 TNBC cell lines. Kong et al. (2014) also reported that IC_50_ of Cucurbitacin E is 10–70 nM in five TNBC cell lines, and among the TNBC cell lines MDA-MB-468 and SW527, Cucurbitacin E significantly decreased cell viability, induced cell cycle G2/M phase arrest, and trigged apoptosis. CuE at a concentration of 0.2 µM decreased the protein levels of CyclinD1, XIAP, Survivin, and Mcl-1 [221].

## 5. Future and Prospects

TNBC is a type of tumor that is aggressive when it comes to metastasis and has a poor prognosis. Medication has a low clinical benefit in TNBC patients, therefore, finding molecular targets for TNBC treatment is essential for acquiring appropriate therapeutic targets. Several pathways can be targeted, including BRCA, Wnt/β-catenin, NF-κB, Hedgehog, JAK, PD-1/PD-L1, PI3K/Akt/m-TOR, EGFR [230,231], and others that have long been part of the treatment strategy. Finding new and effective treatment options for TNBC remains a critical clinical need. To enhance TNBC survival and treatment, a greater understanding of the molecular basis of heterogeneity and the development of improved therapeutic strategies are necessary.

The Wnt/β-catenin signaling pathway is one of the molecular targets in TNBC [232] that is currently being investigated. Wnt activation can cause β-catenin accumulation in the nucleus. It can activate TCF/LEF-1, which promotes the transcription of target genes, thus a molecular understanding of the Wnt/β-catenin pathway is required for suppressing the metastatic pathway in TNBC. To develop more effective drugs, new experimental approaches should be tested in patients with TNBC. Several approaches to TNBC therapy include targeted DNA repair (platinum compounds and taxanes) [233], p53 (taxanes) [234], cell proliferation (anthracycline-containing regimens) [235], and targeted therapy. The best adjuvant regimen for TNBC is still being developed [235].

**Figure 4:**
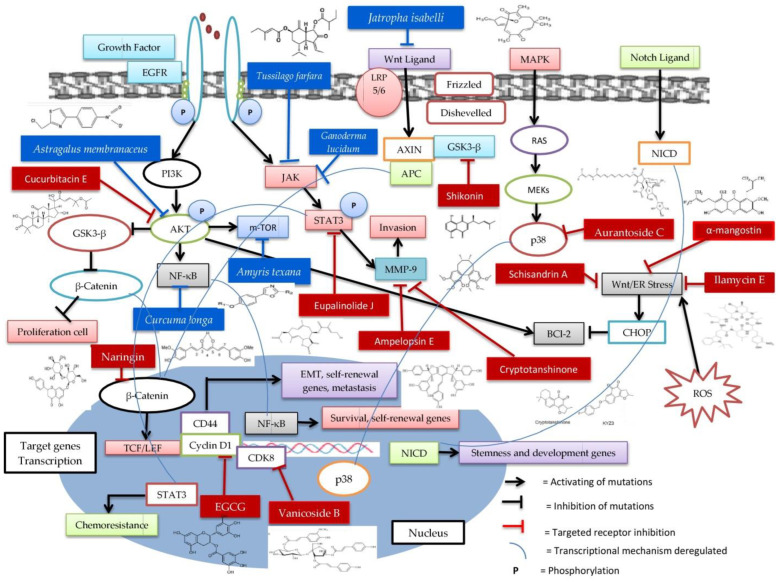
The role of bioactive compounds (red box) and plant extracts (blue box) to inhibit targets involved in TNBC regulation.

As shown in Figure 4, several signaling pathways are associated with genetic mutations in cancer, including the upregulation of the Wnt pathway and growth factors such as EGFR leading to cancer growth, metastasis, cell proliferation, invasion, differentiation, angiogenesis, and apoptosis. Inhibition of the Wnt pathway by inhibitors (bioactive compounds or plant extracts) begins when the Wnt ligand binds to the frizzled main receptor (FZD) and co-receptor LRP5/6 (LRP). The binding of the Wnt ligand to its receptor during signaling leads to disruption of LRP phosphorylation by inhibitors (red and blue boxes) and disheveled inactivation. As a result, the action of AXIN and APC to bind to LRP GSK-3β is inhibited and β-catenin is retained because it is not phosphorylated by GSK-3β. This causes the inactivation of TCF/LEF gene transcription. Inhibition of PI3K/Akt/m-TOR and JAK/STAT3 by bioactive compounds or plant extracts results in the prevention of cell proliferation, invasion, and survival in the EGFR growth pathway. Thus, the degradation of cyclin D1 occurs after being induced by bioactive compounds to encourage the inactivation of Wnt/β-catenin.

Natural substances may be useful in the treatment of breast cancer. Based on previous studies, we resumed the involvement of bioactive compounds and plant extracts to inhibit targets involved in TNBC regulation as shown in Figure 4. In this review, we only explored the natural compounds from extracts and isolates that have an effect on TNBC cell lines in in vivo, in vitro, and in silico studies. Other compounds from natural products are still needed for TNBC treatment agents, thus further developments should be carried out using compounds such as luteolin, α-mangostin, piperine, silibinine, apigenin, quercetin, fisetin, resveratrol, genistein, 10-gingerol, chalcones, berberine, curcumin, epigallocatechin gallate, cyanidin-3-o-glucoside, and glycyrrhizin. The potential oncogenic molecular pathways in TNBCs were discussed, as well as how the dose and purified plant-derived natural compounds selectively target and modify the genes and/or proteins implicated in these aberrant mechanisms to demonstrate anti-cancer potential. The mechanism of action of each natural compound component varies according to the influence of dose, purity, and isolation. Furthermore, the IC_50_ value of natural compounds that inhibits TNBC also influences their mechanisms. One of the chemical components having the potential as a TNBC therapeutic agent is α-mangostin isolated from the mangosteen rind (*Garcinia mangostana* L.). α-mangostin has been shown to suppress the MDA-MB-231 and TNBC cell lines, as well as tumor development and metastasis in a mouse breast cancer model. Therefore, understanding the characteristics of TNBC is critical in identifying effective treatment targets for the development of aggressive TNBC, particularly the metastatic route via the Wnt/β-catenin pathway.

## 6. Conclusions

Chemotherapy or radiotherapy is a common treatment for triple-negative breast cancer; however, it has various side effects such as the occurrence of resistance, narrow therapeutic index, and unselective action of anti-cancer drugs damaging the DNA of cancer cells and regular cells. Numerous studies are being conducted to develop new medicines that are more effective against cancer cells and have fewer adverse effects. Exploring molecules derived from natural sources as anti-cancer treatment agents is one of the potential research avenues. This has led to an alternative therapeutic approach for TNBC using natural compounds.

Many plant-derived natural compounds, including luteolin, α-mangostin, piperine, silibinin, apigenin, quercetin, fisetin, resveratrol, genistein, 10-gingerol, chalcones, berberine, curcumin, epigallocatechin gallate, cyanidin-3-o-glucoside, and glycyrrhizin, have shown anti-cancer properties, especially in the treatment of TNBCs. These compounds exhibit the capability to suppress cell growth, migration, and metastasis by targeting irregular/irregular signaling pathways present in TNBC, such as Wnt/β-Catenin, NF-κB, PI3K/Akt/mTOR, PD-1/PD-L1, LAG-3, CTLA-4, STAT-3, EGFR, Trop-2, RAF/MEK/ERK, JAK, Glycoprotein NMB (GpNMB), and hedgehog pathways. Despite the fact that the natural molecule shows potential against TNBC cell lines, compounds derived from natural resources are currently limited in their usage as TNBC therapeutic agents. Further research on the composition of substances derived from natural resources is needed to determine potential therapeutic candidates and histological characteristics. Data from these studies could provide insight into potential sources of natural compounds that could be used against the aggressive TNBC cells, particularly the metastatic pathway, in a targeted and effective manner.

## Figures and Tables

**Table 1:** Molecular classification of triple-negative breast cancer and ongoing clinical potential therapies in vitro adapted with permission from Lehmann et al. 2014 [41] and Ahn et al. 2016 [14].

TNBC Subtype	Cell Lines	Intrinsic Subtype	Expression of Gene	Potential Therapies
BL1 (Basal like-1)	HCC2157 HCC1599 HCC1937 HCC1143 HCC3153 MDA-MB-468 HCC38 HCC2185	Basal A Basal A HER2 Basal A Basal A Basal A Unclassified/Basal B Basal A	Cell cycle DNA damage response (ATR-BRCA pathway)	PARP inhibitors, Platinum agents [42], Pan-HDAC inhibitor, Wnt/ β-Catenin inhibitor
BL2 (Basal like-2)	SUM149PT CAL851 HCC70 HCC1806 HDQ-P1 HCC1500	Unclassified/Basal B Basal A Basal Unclassified/Basal A Unclassified Basal B	Growth factor Signaling pathways (EGFR, MET, NGF, Wnt/β-Catenin, IGF-IR) Glycolysis, Gluconeogenesis	PARP inhibitors, Platinum agents [42,43], mTOR inhibitors Growth-factor inhibitors [44], Wnt/ β-Catenin inhibitor
IM (Immunomodulatory)	HCC1187 DU4475	Basal A Unclassified	Immune signaling (CTLA4, ILI 2, IL7 pathways antigen processing/presentation) cytokine signaling by JAK/STAT, TNF, and NF-κB pathways	(PD1/PD-L1 inhibitors, CTLA-4 inhibitor, LAG-3 inhibitor, Anti TIM-3 mAb, Hedgehog inhibitor) [14,42]
M (Mesenchymal like)	BT-549 CAL-51 CAL-120	Unclassified/Basal B Unclassified Luminal B	EMT Growth factor signaling Cell motility Cell differentiation	Tyrosine kinase inhibitors PI3K/mTOR inhibitors EMT and CSC targeted MET inhibitor FGFR, EGFR, VEGFR inhibitor [14,41,42]
MSL (Mesenchymal Stem Cell-like)	Hs578T MDA-MB-157 SUM159PT MDA-MB-436 MDA-MB-231	Unclassified/Basal B Unclassified/Basal B Unclassified/Basal B Unclassified/Basal B Unclassified/Basal B	EMT Growth factor Proliferation (decreased) Angiogenesis genes	Tyrosine kinase inhibitors PI3K/mTOR inhibitors Antiangiogenic Src antagonist MET inhibitor, Trop-2 inhibitor [14,41,45]
LAR (Luminal Androgen Receptor)	MDA-MB-453 HCC2185 CAL-14 SUM185PE MFM-223	Luminal A Luminal A Luminal A Luminal A Luminal A/B	Androgen Receptor Luminal gene expression pattern Molecular apocrine subtype	Androgen Receptor targeted PI3K inhibitors [41,42]
Unclassified	HCC1395 BT20 SW527	Basal HER2/Basal A Luminal B	-	-

Abbreviations: Cytotoxic T lymphocyte-associated protein 4 (CTLA-4); epithelial–mesenchymal transition (EMT); epidermal growth factor receptor (EGFR); fibroblast growth factor receptor (FGFR); histone deacetylase (HDAC); human epidermal growth factor receptor 2 (HER2); Janus kinase (JAK); lymphocyte-activation gene 3 (LAG-3); mechanistic target of rapamycin (mTOR); programmed cell death protein 1 (PD-1); programmed death-ligand (PD-L1); poly-ADP ribose polymerase (PARP); phosphoinositide 3-kinase (PI3K); T-cell immunoglobulin and mucin-domain containing-3 (TIM-3); trophoblast antigen 2 (Trop-2); vascular endothelial growth factor receptor (VEGFR).

**Table 2:** Natural compound’s activity in cell line and its mechanism.

Natural Product	Cell Lines	Mechanism	Methods	Reference
Ilamycin E (*Streptomyces atratus*) Actinomycetes	HCC1937 and MDA-MB-468	Inhibition of endoplasmic reticulum (ER) stress and CHOP-BCl2	In vitro	[202]
Schisandrin A	MDA-MB-231	Inhibition of Wnt/ER stress	In vitro and in vivo (Xenograft mouse)	[203]
Ampelopsin E, Oligostilbene (Dryobalanops)	MDA-MB-231	Inhibition of invadopodia formation by stopping migration, transmigration, and invasive expressions of PDGF MMP2, MMP9, MMP14	In vitro	[204]
Aurantoside C (C828) (Sponge *Manihinealynbeazleyae*)	MDA-MB-231, SUM159PT and SUM149	Inhibition of the phosphorylation of Akt/mTORdan NF-κB pathways and increased the phosphorylation of p38 MAPK and SAPK/JNK pathway	In vitro	[205]
*Amyris texana* (Oxazole) Discovery of Compound 30 (CIDD-0067106)	MDA-MB-453	Inhibition of the activity of the mTORC1 pathway, a model of the Luminal Androgen Receptor (LAR)	In vitro and in silico	[206]
A sequesterpenoid from *Farfarae Flos* (*Tussilago farfara*)	MDA-MB-231	Inhibition of JAK-STAT3 signaling	In vitro and in vivo (Tumor Xenograft)	[207]
Diterpen Jatrophone (*Jatropha isabelli*)	MDA-MB-231, HCC38, MDA-MB-157 and MDA-MB-468	Inhibition of Wnt/β-Catenin signaling and proliferation and EMT	In vitro	[208,209]
Naringin/Flavonoid (*Dynaria fortunei, citrus aurantium, citrus medica* L.)	MDA-MB-231	Inhibition of growth potential by targeting β-Catenin signaling pathway	In vitro and in vivo (Xenograft mice)	[210]
*Myrothamnus flabelli* folius (Derivative of Galloyl glucose hexahydroxydiphenic acid)	BT-549T and MDA-MB-231	Inhibited the growth cell	In vitro	[211]
Cryptotanshinone (*Salviamiltiorrhiza* Bunge)	MDA-MB-231	Inhibition KYZ3 by decreasing the level of MMP-9 with activated STAT3	In vitro, in silico, and in vivo (Subcutaneous implantation),	[212]
*Curcuma longa*	SUM149 and MDA-MB-231	Inhibition of NF-κB transcriptional factor activity and consequently the expression of some NF-κB targets	In vitro	[213]
*Ganoderma lucidum*	SUM149 and MDA-MB-231	Inhibition of STAT3 and JAK2	In vitro and in vivo (Injected limiting dilutions combined immunodeficient (CD44+/CD24–)	[214]
*Annonamuricata* leaf	MDA-MB-231	Intrinsic Apoptotic pathway	In vitro	[158]
Shikonin (*Lithospermum erythrorhizon* Sieb. et Zucc)	MDA-MB-231 and 4T1	Inhibition of the epithelial-to-mesenchymal transition via glycogen synthase kinase 3β-regulated suppression of β-catenin signaling	In vitro	[215]
*Astragalus membranaceus*	MDA-MB-231	Inhibition of PIK3CG/AKT/BCL2 signaling pathway	In vitro and in silico	[216]
Vanicoside B (*Persicaria dissitiflora*)	MDA-MB-231 and HCC38	Inhibition CDK8-signaling pathway	In vitro and in vivo (Tumor Xenograft Model)	[217]
Eupalinolide J (*Eupatorium lindleyanum* DC)	MDA-MB-231 and MDA-MB-468	Suppressing growth by STAT3 signaling pathways such as anti-apoptosis, cell cycle arrest, and MMP disruption	In vitro and in vivo (Xenograft Mouse Model)	[218]
Cantharidin Component of terpenoidsecreted by the blister beetle *Mylabris phalerata* (Pallas)	MDA-MB-231 and MDA-MB-468	Suppressing Autophagy and Inducing apoptosis by inhibiting the conversion of LC3 I to LC3 II and suppressing the expression of Beclin-1	In vitro and in vivo (Subcutaneous inoculation)	[219]
Benzimidazole compounds (SRI33576 and SRI35889)	MDA-MB-231 and MDA-MB-468	Inhibition of Wnt/β-Catenin signaling and also detract of mTOR, STAT3 and Notch signaling	In vitro	[220]
Cucurbitacin E from *Hemsleya delavayi* var. yalungensis (Cucurbitaceae)	MDA-MB-468 and SW527	Induced cell cycle G2/M phase arrest and apoptosis by expression of Cyclin D1, Survivin, XIAP, Bcl2, and Mcl-1 and increased activation of JNK and inhibited activation of AKT and ERK within MDA-MB-468	In vitro	[221]
α-mangostin (*Garcinia mangostana* L.)	MDA-MB-231 and MCF-7	Induced endoplasmic reticulum stress and autophagy by fatty acid synthase inhibition mediated apoptosis	In vitro	[222]

## Data Availability

Not applicable.

## Abbreviations

Activated serin-threonin protein kinase (AKT); adenomatous polyposis coli (APC); aldehyde dehydrogenase 1 (ALDH1); ataxia telangiectasia (ATR); ATP-binding cassette transporter G2 (ABCG2); human B-cell lymphoma (BCL-2); C/EBP homologous protein (CHOP); chemokine (C-C motif) ligand 2 (CCL2); connective tisue growth factor (CTGF); cysteine-rich angiogenic inducer 61 (CYR61); cytotoxic T lymphocyte-associated protein 4 (CTLA-4); hepatocellular carcinoma deletion gene 1 (DLC1); epigallocatechin gallate (EGCG); epithelial–mesenchymal transition (EMT); epidermal growth factor receptor (EGFR); extracellular signal regulated kinase (ERK); enhancer of zeste homolog 2 (EZH2); extracellular signal regulated kinase (ERK); fatty acid synthase (FASN); fibroblast growth factor receptor (FGFR); endoplasmic reticulum stress (ER stress); growth arrest and DNA damage 45 (GADD45); glycogen synthase kinase 3 beta (GSK3-β); histone deacetylase (HDAC); human epidermal growth factor receptor 2 (HER2); Janus kinase (JAK); microtubule-associated protein light chain 3 (LC3); lymphocyte-activation gene 3 (LAG-3); mechanistic target of rapamycin (mTOR); mitogen-activated protein kinase (MAPK); mitogen extracellular signal regulated kinase (MEK); matrix metalloproteinase-9 (MMP-9); messenger ribonucleic acid (mRNA); myeloid cell leukemia-1 (Mcl-1); notch intracellular domain (NICD); nuclear factor erythroid 2- related factor 2 (Nrf2); nuclear factor-kappaBeta (NF-kB); programmed cell death protein 1 (PD-1); programmed death-ligand (PD-L1); poly-ADP ribose polymerase (PARP); phosphoinositide 3-kinase (PI3K); phosphorylation (P); reactive oxygen species (ROS); T-cell factor/lymphoid enhancer factor (TCF/LEF); rat sarcoma (RAS); stress-activated protein kinases (SAPK); trascriptional coactivator with PDZ-binding motif (TAZ); T-cell immunoglobulin and mucin-domain containing-3 (TIM-3); thin layer chromatography (TLC); trophoblast antigen 2 (Trop-2); vascular endothelial growth factor receptor (VEGFR); wingless signaling pathway (WNT); X-linked inhibitor of apoptosis protein (XIAP); yes-associated protein (YAP).

## References

[1] Anastasiadi Z., Lianos G.D., Ignatiadou E., Harissis H.V., Mitsis M. (2017). Breast cancer in young women: An overview. Updates Surg..

[2] Li Y., Zhan Z., Yin X., Fu S., Deng X. (2021). Targeted Therapeutic Strategies for Triple-Negative Breast Cancer. Front. Oncol..

[3] Metzger-Filho O., Tutt A., de Azambuja E., Saini K.S., Viale G., Loi S., Bradbury I., Bliss J.M., Azim H.A., Ellis P. (2012). Dissecting the heterogeneity of triple-negative breast cancer. J. Clin. Oncol..

[4] Wu Q., Siddharth S., Sharma D. (2021). Triple Negative Breast Cancer: A Mountain Yet to Be Scaled Despite the Triumphs. Cancers.

[5] Jin J., Gao Y., Zhang J., Wang L., Wang B., Cao J., Shao Z., Wang Z. (2018). Incidence, pattern and prognosis of brain metastases in patients with metastatic triple negative breast cancer. BMC Cancer.

[6] Yao Y., Chu Y., Xu B., Hu Q., Song Q. (2019). Risk factors for distant metastasis of patients with primary triple-negative breast cancer. Biosci. Rep..

[7] Fares J., Fares M.Y., Khachfe H.H., Salhab H.A., Fares Y. (2020). Molecular principles of metastasis: A hallmark of cancer revisited. Signal Transduct. Target. Ther..

[8] Marino N., Woditschka S., Reed L.T., Nakayama J., Mayer M., Wetzel M., Steeg P.S. (2013). Breast cancer metastasis: Issues for the personalization of its prevention and treatment. Am. J. Pathol..

[9] Gupta G.K., Collier A.L., Lee D., Hoefer R.A., Zheleva V., Siewertsz van Reesema L.L., Tang-Tan A.M., Guye M.L., Chang D.Z., Winston J.S. (2020). Perspectives on Triple-Negative Breast Cancer: Current Treatment Strategies, Unmet Needs, and Potential Targets for Future Therapies. Cancers.

[10] El Sayed R., El Jamal L., El Iskandarani S., Kort J., Abdel Salam M., Assi H. (2019). Endocrine and targeted therapy for hormone-receptor-positive, HER2-negative advanced breast cancer: Insights to sequencing treatment and overcoming resistance based on clinical trials. Front. Oncol..

[11] Al-Mahmood S., Sapiezynski J., Garbuzenko O.B., Minko T. (2018). Metastatic and triple-negative breast cancer: Challenges and treatment options. Drug Deliv. Transl. Res..

[12] Gámez-Pozo A., Trilla-Fuertes L., Prado-Vázquez G., Chiva C., López-Vacas R., Nanni P., Berges-Soria J., Grossmann J., Díaz-Almirón M., Ciruelos E. (2017). Prediction of adjuvant chemotherapy response in triple negative breast cancer with discovery and targeted proteomics. PLoS ONE.

[13] Yao Y., Chu Y., Xu B., Hu Q., Song Q. (2019). Radiotherapy after surgery has significant survival benefits for patients with triple-negative breast cancer. Cancer Med..

[14] Ahn S.G., Kim S.J., Kim C., Jeong J. (2016). Molecular classification of triple-negative breast cancer. J. Breast Cancer.

[15] Hubalek M., Czech T., Müller H. (2017). Biological Subtypes of Triple-Negative Breast Cancer. Breast Care.

[16] Hirshfield K.M., Ganesan S. (2014). Triple-negative breast cancer: Molecular subtypes and targeted therapy. Curr. Opin. Obstet. Gynecol..

[17] Ashraf M.A. (2020). Phytochemicals as potential anticancer drugs: Time to ponder nature’s bounty. BioMed Res. Int..

[18] Saldanha S.N., Tollefsbol T.O. (2012). The role of nutraceuticals in chemoprevention and chemotherapy and their clinical outcomes. J. Oncol..

[19] De P., Carlson J.H., Wu H., Marcus A., Leyland-Jones B., Dey N. (2016). Wnt-beta-catenin pathway signals metastasis-associated tumor cell phenotypes in triple negative breast cancers. Oncotarget.

[20] Zhu H., Bhaijee F., Ishaq N., Pepper D.J., Backus K., Brown A.S., Zhou X., Miele L. (2013). Correlation of Notch1, pAKT and nuclear NF-κB expression in triple negative breast cancer. Am. J. Cancer Res..

[21] Costa R.L., Han H.S., Gradishar W.J. (2018). Targeting the PI3K/AKT/mTOR pathway in triple-negative breast cancer: A review. Breast Cancer Res. Treat..

[22] Santoni M., Romagnoli E., Saladino T., Foghini L., Guarino S., Capponi M., Giannini M., Cognigni P.D., Ferrara G., Battelli N. (2018). Triple negative breast cancer: Key role of tumor-associated macrophages in regulating the activity of anti-PD-1/PD-L1 agents. Biochim. Biophys. Acta-Rev. Cancer.

[23] Stovgaard E.S., Kümler I., List-Jensen K., Roslind A., Christensen I.J., Høgdall E., Nielsen D., Balslev E. (2021). Prognostic and Clinicopathologic Associations of LAG-3 Expression in Triple-negative Breast Cancer. Appl. Immunohistochem. Mol. Morphol..

[24] Peng Z., Su P., Yang Y., Yao X., Zhang Y., Jin F., Yang B. (2020). Identification of CTLA-4 associated with tumor microenvironment and competing interactions in triple negative breast cancer by co-expression network analysis. J. Cancer.

[25] McDaniel J.M., Varley K.E., Gertz J., Savic D.S., Roberts B.S., Bailey S.K., Shevde L.A., Ramaker R.C., Lasseigne B.N., Kirby M.K. (2017). Genomic regulation of invasion by STAT3 in triple negative breast cancer. Oncotarget.

[26] El Guerrab A., Bamdad M., Kwiatkowski F., Bignon Y.-J., Penault-Llorca F., Aubel C. (2016). Anti-EGFR monoclonal antibodies and EGFR tyrosine kinase inhibitors as combination therapy for triple-negative breast cancer. Oncotarget.

[27] Khoury K., Feldman R., Pohlmann P.R., Heeke A.L., Gatalica Z., Veloso Y., Vidal G.A., Schwartzberg L.S., Swain S.M., Isaacs C. (2019). Molecular characterization of trophoblast cell surface antigen 2 (Trop-2) positive triple negative breast cancer (TNBC). J. Clin. Oncol..

[28] Nagaria T.S., Shi C., Leduc C., Hoskin V., Sikdar S., Sangrar W., Greer P.A. (2017). Combined targeting of Raf and Mek synergistically inhibits tumorigenesis in triple negative breast cancer model systems. Oncotarget.

[29] Song X., Liu Z., Yu Z. (2020). EGFR promotes the development of triple negative breast cancer through JAK/STAT3 Signaling. Cancer Manag. Res..

[30] Huang Y.-H., Chu P.-Y., Chen J.-L., Huang C.-T., Huang C.-C., Tsai Y.F., Wang Y.-L., Lien P.-J., Tseng L.-M., Liu C.-Y. (2021). Expression pattern and prognostic impact of glycoprotein non-metastatic B (GPNMB) in triple-negative breast cancer. Sci. Rep..

[31] Habib J.G., O’Shaughnessy J.A. (2016). The hedgehog pathway in triple-negative breast cancer. Cancer Med..

[32] Varghese E., Samuel S.M., Abotaleb M., Cheema S., Mamtani R., Büsselberg D. (2018). The “Yin and Yang” of natural compounds in anticancer therapy of triple-negative breast cancers. Cancers.

[33] Yang Z., Zhang Q., Yu L., Zhu J., Cao Y., Gao X. (2020). The signaling pathways and targets of traditional Chinese medicine and natural medicine in triple-negative breast cancer. J. Ethnopharmacol..

[34] Thakuri P.S., Gupta M., Singh S., Joshi R., Glasgow E., Lekan A., Agarwal S., Luker G.D., Tavana H. (2020). Phytochemicals inhibit migration of triple negative breast cancer cells by targeting kinase signaling. BMC Cancer.

[35] Kurubanjerdjit N. (2020). Identifying the regulation mechanism of phytochemicals on triple negative breast cancer’s biological network. Gene Rep..

[36] Malla R.R., Deepak K., Merchant N., Dasari V.R. (2020). Breast Tumor Microenvironment: Emerging target of therapeutic phytochemicals. Phytomedicine.

[37] Ossovskaya V., Wang Y., Budoff A., Xu Q., Lituev A., Potapova O., Vansant G., Monforte J., Daraselia N. (2011). Exploring Molecular Pathways of Triple-Negative Breast Cancer. Genes Cancer.

[38] Chavez K.J., Garimella S.V., Lipkowitz S. (2010). Triple negative breast cancer cell lines: One tool in the search for better treatment of triple negative breast cancer. Breast Dis..

[39] Anders C.K., Zagar T.M., Carey L.A. (2013). The management of early-stage and metastatic triple-negative breast cancer: A review. Hematol. Oncol. Clin. N. Am..

[40] Ensenyat-Mendez M., Llinàs-Arias P., Orozco J.I.J., Íñiguez-Muñoz S., Salomon M.P., Sesé B., DiNome M.L., Marzese D.M. (2021). Current Triple-Negative Breast Cancer Subtypes: Dissecting the Most Aggressive Form of Breast Cancer. Front. Oncol..

[41] Lehmann B.D., Jovanović B., Chen X., Estrada M.V., Johnson K.N., Shyr Y., Moses H.L., Sanders M.E., Pietenpol J.A. (2016). Refinement of triple-negative breast cancer molecular subtypes: Implications for neoadjuvant chemotherapy selection. PLoS ONE.

[42] Lehmann B.D., Pietenpol J.A. (2014). Identification and use of biomarkers in treatment strategies for triple-negative breast cancer subtypes. J. Pathol..

[43] Jhan J.-R., Andrechek E.R. (2017). Triple-negative breast cancer and the potential for targeted therapy. Pharmacogenomics.

[44] He J., McLaughlin R.P., van der Noord V., Foekens J.A., Martens J.W., van Westen G., Zhang Y., van de Water B. (2019). Multi-targeted kinase inhibition alleviates mTOR inhibitor resistance in triple-negative breast cancer. Breast Cancer Res. Treat..

[45] Masuda H., Baggerly K.A., Wang Y., Zhang Y., Gonzalez-Angulo A.M., Meric-Bernstam F., Valero V., Lehmann B.D., Pietenpol J.A., Hortobagyi G.N. (2013). Differential response to neoadjuvant chemotherapy among 7 triple-negative breast cancer molecular subtypes. Clin. Cancer Res..

[46] Lehmann B.D., Bauer J.A., Schafer J.M., Pendleton C.S., Tang L., Johnson K.C., Chen X., Balko J.M., Gómez H., Arteaga C.L. (2014). PIK3CA mutations in androgen receptor-positive triple negative breast cancer confer sensitivity to the combination of PI3K and androgen receptor inhibitors. Breast Cancer Res..

[47] Bianchini G., Balko J.M., Mayer I.A., Sanders M.E., Gianni L. (2016). Triple-negative breast cancer: Challenges and opportunities of a heterogeneous disease. Nat. Rev. Clin. Oncol..

[48] Jézéquel P., Kerdraon O., Hondermarck H., Guérin-Charbonnel C., Lasla H., Gouraud W., Canon J.L., Gombos A., Dalenc F., Delaloge S. (2019). Identification of three subtypes of triple-negative breast cancer with potential therapeutic implications. Breast Cancer Res..

[49] Andre F., Zielinski C. (2012). Optimal strategies for the treatment of metastatic triple-negative breast cancer with currently approved agents. Ann. Oncol..

[50] Collignon J., Lousberg L., Schroeder H., Jerusalem G. (2016). Triple-negative breast cancer: Treatment challenges and solutions. Breast Cancer.

[51] Kim C., Gao R., Sei E., Brandt R., Hartman J., Hatschek T., Crosetto N., Foukakis T., Navin N.E. (2018). Chemoresistance Evolution in Triple-Negative Breast Cancer Delineated by Single-Cell Sequencing. Cell.

[52] Pardoll D.M. (2012). The blockade of immune checkpoints in cancer immunotherapy. Nat. Rev. Cancer.

[53] Soliman H., Khalil F., Antonia S. (2014). PD-L1 expression is increased in a subset of basal type breast cancer cells. PLoS ONE.

[54] Nakhjavani M., Hardingham J.E., Palethorpe H.M., Price T.J., Townsend A.R. (2019). Druggable molecular targets for the treatment of triple negative breast cancer. J. Breast Cancer.

[55] Selby M.J., Engelhardt J.J., Quigley M., Henning K.A., Chen T., Srinivasan M., Korman A.J. (2013). Anti-CTLA-4 antibodies of IgG2a isotype enhance antitumor activity through reduction of intratumoral regulatory T cells. Cancer Immunol. Res..

[56] Vargas F.A., Furness A.J.S., Litchfield K., Joshi K., Rosenthal R., Ghorani E., Solomon I., Lesko M.H., Ruef N., Roddie C. (2018). Fc Effector Function Contributes to the Activity of Human Anti-CTLA-4 Antibodies. Cancer Cell.

[57] Buchbinder E.I., Desai A. (2016). CTLA-4 and PD-1 pathways similarities, differences, and implications of their inhibition. Am. J. Clin. Oncol..

[58] Vikas P., Borcherding N., Zhang W. (2018). The clinical promise of immunotherapy in triple-negative breast cancer. Cancer Manag. Res..

[59] Qin S., Xu L., Yi M., Yu S., Wu K., Luo S. (2019). Novel immune checkpoint targets: Moving beyond PD-1 and CTLA-4. Mol. Cancer.

[60] Harris L.G., Pannell L.K., Singh S., Samant R.S., Shevde L.A. (2012). Increased vascularity and spontaneous metastasis of breast cancer by hedgehog signaling mediated upregulation of cyr61. Oncogene.

[61] Colavito S.A., Zou M.R., Yan Q., Nguyen D.X., Stern D.F. (2014). Significance of glioma-associated oncogene homolog 1 (GLI1) expression in claudin-low breast cancer and crosstalk with the nuclear factor kappa-light-chain-enhancer of activated B cells (NFκB) pathway. Breast Cancer Res..

[62] Riobo N.A., Haines G.M., Emerson C.P. (2006). Protein kinase C-δ and mitogen-activated protein/extracellular signal–regulated kinase-1 control GLI activation in Hedgehog signaling. Cancer Res..

[63] Goel H.L., Pursell B., Chang C., Shaw L.M., Mao J., Simin K., Kumar P., Vander Kooi C.W., Shultz L.D., Greiner D.L. (2013). GLI1 regulates a novel neuropilin-2/α6β1 integrin based autocrine pathway that contributes to breast cancer initiation. EMBO Mol. Med..

[64] Duan Z.H., Wang H.C., Zhao D.M., Ji X.X., Song M., Yang X.J., Cui W. (2015). Cooperatively transcriptional and epigenetic regulation of sonic hedgehog overexpression drives malignant potential of breast cancer. Cancer Sci..

[65] Tacchini L., de Ponti C., Matteucci E., Follis R., Desiderio M.A. (2004). Hepatocyte growth factor-activated NF-κB regulates HIF-1 activity and ODC expression, implicated in survival, differently in different carcinoma cell lines. Carcinogenesis.

[66] Wang Y., Shen Y., Wang S., Shen Q., Zhou X. (2018). The role of STAT3 in leading the crosstalk between human cancers and the immune system. Cancer Lett..

[67] Dhillon K.K., Swisher E.M., Taniguchi T. (2011). Secondary mutations of BRCA1/2 and drug resistance. Cancer Sci..

[68] Catana A., Apostu A.P., Antemie R.G. (2019). Multi gene panel testing for hereditary breast cancer—Is it ready to be used?. Med. Pharm. Rep..

[69] Couch F.J., Hart S.N., Sharma P., Toland A.E., Wang X., Miron P., Olson J.E., Godwin A.K., Pankratz V.S., Olswold C. (2015). Inherited mutations in 17 breast cancer susceptibility genes among a large triple-negative breast cancer cohort unselected for family history of breast cancer. J. Clin. Oncol..

[70] Kim M.P., Zhang Y., Lozano G. (2015). Mutant p53: Multiple mechanisms define biologic activity in cancer. Front. Oncol..

[71] Sandhu S.K., Yap T.A., de Bono J.S. (2011). The Emerging Role of Poly(ADP-Ribose) Polymerase Inhibitors in Cancer Treatment. Curr. Drug Targets.

[72] Davar D., Beumer J.H., Hamieh L., Tawbi H. (2012). Role of PARP Inhibitors in Cancer Biology and Therapy. Curr. Med. Chem..

[73] Livraghi L., Garber J.E. (2015). PARP inhibitors in the management of breast cancer: Current data and future prospects. BMC Med..

[74] Kaufman B., Shapira-Frommer R., Schmutzler R.K., Audeh M.W., Friedlander M., Balmaña J., Mitchell G., Fried G., Stemmer S.M., Hubert A. (2015). Olaparib monotherapy in patients with advanced cancer and a germline BRCA1/2 mutation. J. Clin. Oncol..

[75] Pahuja S., Beumer J.H., Appleman L.J., Tawbi H.A.-H., Stoller R.G., Lee J.J., Lin Y., Ding F., Yu J., Belani C.P. (2015). A phase I study of veliparib (ABT-888) in combination with weekly carboplatin and paclitaxel in advanced solid malignancies and enriched for triple-negative breast cancer (TNBC). J. Clin. Oncol..

[76] New M., Olzscha H., La Thangue N.B. (2012). HDAC inhibitor-based therapies: Can we interpret the code?. Mol. Oncol..

[77] Garmpis N., Damaskos C., Garmpi A., Kalampokas E., Kalampokas T., Spartalis E., Daskalopoulou A., Valsami S., Kontos M., Nonni A. (2017). Histone deacetylases as new therapeutic targets in triple-negative breast cancer: Progress and promises. Cancer Genom. Proteom..

[78] Olzscha H., Sheikh S., La Thangue N.B. (2015). Deacetylation of chromatin and gene expression regulation: A new target for epigenetic therapy. Crit. Rev. Oncog..

[79] Tate C.R., Rhodes L.V., Segar H.C., Driver J.L., Pounder F.N., Burow M.E., Collins-Burow B.M. (2012). Targeting triple-negative breast cancer cells with the histone deacetylase inhibitor panobinostat. Breast Cancer Res..

[80] Slingerland M., Guchelaar H.J., Gelderblom H. (2014). Histone deacetylase inhibitors: An overview of the clinical studies in solid tumors. Anti-Cancer Drugs.

[81] Schech A., Kazi A., Yu S., Shah P., Sabnis G. (2015). Histone deacetylase inhibitor entinostat inhibits tumor-initiating cells in triple-negative breast cancer cells. Mol. Cancer Ther..

[82] Merino V.F., Nguyen N., Jin K., Sadik H., Cho S., Korangath P., Han L., Foster Y.M.N., Zhou X.C., Zhang Z. (2016). Combined treatment with epigenetic, differentiating, and chemotherapeutic agents cooperatively targets tumor-initiating cells in triple-negative breast cancer. Cancer Res..

[83] Kai M., Kanaya N., Wu S.V., Mendez C., Nguyen D., Luu T., Chen S. (2015). Targeting breast cancer stem cells in triple-negative breast cancer using a combination of LBH589 and salinomycin. Breast Cancer Res. Treat..

[84] Mrklić I., Pogorelić Z., Ćapkun V., Tomić S.Ž. (2013). Expression of androgen receptors in triple negative breast carcinomas. Acta Histochem..

[85] Astvatsaturyan K., Yue Y., Walts A.E., Bose S. (2018). Androgen receptor positive triple negative breast cancer: Clinicopathologic, prognostic, and predictive features. PLoS ONE.

[86] Pietri E., Conteduca V., Andreis D., Massa I., Melegari E., Sarti S., Cecconetto L., Schirone A., Bravaccini S., Serra P. (2016). Androgen receptor signaling pathways as a target for breast cancer treatment. Endocr. Relat. Cancer.

[87] Gerratana L., Basile D., Buono G., de Placido S., Giuliano M., Minichillo S., Coinu A., Martorana F., de Santo I., Del Mastro L. (2018). Androgen receptor in triple negative breast cancer: A potential target for the targetless subtype. Cancer Treat. Rev..

[88] He J., Peng R., Yuan Z., Wang S., Peng J., Lin G., Jiang X., Qin T. (2012). Prognostic value of androgen receptor expression in operable triple-negative breast cancer: A retrospective analysis based on a tissue microarray. Med. Oncol..

[89] Caiazza F., Murray A., Madden S.F., Synnott N.C., Ryan E.J., O’Donovan N., Crown J., Duffy M.J. (2016). Preclinical evaluation of the AR inhibitor enzalutamide in triple-negative breast cancer cells. Endocr. Relat. Cancer.

[90] Asano Y., Kashiwagi S., Goto W., Tanaka S., Morisaki T., Takashima T., Noda S., Onoda N., Ohsawa M., Hirakawa K. (2017). Expression and clinical significance of androgen receptor in triple-negative breast cancer. Cancers.

[91] Hu R., Dawood S., Holmes M.D., Collins L.C., Schnitt S.J., Cole K., Marotti J.D., Hankinson S.E., Colditz G.A., Tamimi R.M. (2011). Androgen receptor expression and breast cancer survival in postmenopausal women. Clin. Cancer Res..

[92] Rahim B., O’Regan R. (2017). AR signaling in breast cancer. Cancers.

[93] Kim Y., Jae E., Yoon M. (2015). Influence of androgen receptor expression on the survival outcomes in breast cancer: A meta-analysis. J. Breast Cancer.

[94] Gucalp A., Tolaney S., Isakoff S.J., Ingle J.N., Liu M.C., Carey L.A., Blackwell K., Rugo H., Nabell L., Forero A. (2013). Phase II trial of bicalutamide in patients with androgen receptor-positive, estrogen receptor-negative metastatic breast cancer. Clin. Cancer Res..

[95] Barton V.N., D’Amato N.C., Gordon M.A., Lind H.T., Spoelstra N.S., Babbs B.L., Heinz R.E., Elias A., Jedlicka P., Jacobsen B.M. (2015). Multiple molecular subtypes of triple-negative breast cancer critically rely on androgen receptor and respond to enzalutamide in vivo. Mol. Cancer Ther..

[96] Traina T.A., Miller K., Yardley D.A., Eakle J., Schwartzberg L.S., O’Shaughnessy J., Gradishar W., Schmid P., Winer E., Kelly C. (2018). Enzalutamide for the treatment of androgen receptor-expressing triple-negative breast cancer. J. Clin. Oncol..

[97] Bardia A., Gucalp A., DaCosta N., Gabrail N., Danso M., Ali H., Blackwell K.L., Carey L.A., Eisner J.R., Baskin-Bey E.S. (2018). Phase 1 study of seviteronel, a selective CYP17 lyase and androgen receptor inhibitor, in women with estrogen receptor-positive or triple-negative breast cancer. Breast Cancer Res. Treat..

[98] Crouch B.T., Gallagher J., Wang R., Duer J., Hall A., Soo M.S., Hughes P., Haystead T., Ramanujam N. (2019). Exploiting heat shock protein expression to develop a non-invasive diagnostic tool for breast cancer. Sci. Rep..

[99] Den R.B., Lu B. (2012). Heat shock protein 90 inhibition: Rationale and clinical potential. Ther. Adv. Med. Oncol..

[100] Caldas-Lopes E., Cerchietti L., Ahn J.H., Clement C.C., Robles A.I., Rodina A., Moulick K., Taldone T., Gozrnan A., Guo Y. (2009). Hsp90 inhibitor PU-H71, a multimodal inhibitor of malignancy, induces complete responses in triple-negative breast cancer models. Proc. Natl. Acad. Sci. USA.

[101] Proverbs-Singh T., Feldman J.L., Morris M.J., Autio K.A., Traina T.A. (2015). Targeting the androgen receptor in prostate and breast cancer: Several new agents in development. Endocr. Relat. Cancer.

[102] Jhaveri K., Chandarlapaty S., Lake D., Gilewski T., Robson M., Goldfarb S., Drullinsky P., Sugarman S., Wasserheit-Leiblich C., Fasano J. (2014). A phase II open-label study of ganetespib, a novel heat shock protein 90 inhibitor for patients with Metastatic breast cancer. Clin. Breast Cancer.

[103] Kou X., Jiang X., Liu H., Wang X., Sun F., Han J., Fan J., Feng G., Lin Z., Jiang L. (2018). Simvastatin functions as a heat shock protein 90 inhibitor against triple-negative breast cancer. Cancer Sci..

[104] Mitri Z., Karakas C., Wei C., Briones B., Simmons H., Ibrahim N., Alvarez R., Murray J.L., Keyomarsi K., Moulder S. (2015). A phase 1 study with dose expansion of the CDK inhibitor dinaciclib (SCH 727965) in combination with epirubicin in patients with metastatic triple negative breast cancer. Invest. New Drugs.

[105] Chien A.J., Rahmaputri S., Dittrich H.F., Majure M.C., Rugo H.S., Melisko M.E., Goga A. (2019). A phase Ib trial of the cyclin-dependent kinase inhibitor dinaciclib (dina) in combination with pembrolizumab (P) in patients with advanced triple-negative breast cancer (TNBC). J. Clin. Oncol..

[106] Paplomata E., O’regan R. (2014). The PI3K/AKT/mTOR pathway in breast cancer: Targets, trials and biomarkers. Ther. Adv. Med. Oncol..

[107] Yang J., Nie J., Ma X., Wei Y., Peng Y., Wei X. (2019). Targeting PI3K in cancer: Mechanisms and advances in clinical trials. Mol. Cancer.

[108] Chan J.J., Tan T.J.Y., Dent R.A. (2019). Novel therapeutic avenues in triple-negative breast cancer: PI3K/AKT inhibition, androgen receptor blockade, and beyond. Ther. Adv. Med. Oncol..

[109] Sharma V., Sharma A.K., Punj V., Priya P. (2019). Recent nanotechnological interventions targeting PI3K/Akt/mTOR pathway: A focus on breast cancer. Semin. Cancer Biol..

[110] van der Noord V.E., McLaughlin R.P., Smid M., Foekens J.A., Martens J.W.M., Zhang Y., van de Water B. (2019). An increased cell cycle gene network determines MEK and Akt inhibitor double resistance in triple-negative breast cancer. Sci. Rep..

[111] Johnson G.L., Amos K.D., Duncan J.S., Whittle M., Zawistowki J., Goulet D., He X., Noe J., Perou C.M., Earp S. (2013). Kinome reprogramming response to MEK inhibition: A window-of-opportunity trial in triple-negative breast cancer (TNBC). J. Clin. Oncol..

[112] Ramaswamy B., Mrozek E., Lustberg M., Wesolowski R., Layman R., Abdel-Rasoul M., Timmers C., Patrick R., Sexton J., Macrae E. Abstract LB-216: NCI 9455: Phase II study of trametinib followed by trametinib plus AKT inhibitor, GSK2141795 in patients with advanced triple negative breast cancer. Proceedings of the AACR 107th Annual Meeting.

[113] Shuai K., Liu B. (2003). Regulation of JAK–STAT signalling in the immune system. Nat. Rev. Immunol..

[114] Balko J.M., Schwarz L.J., Luo N., Estrada M.V., Giltnane J.M., Dávila-González D., Wang K., Sánchez V., Dean P.T., Combs S.E. (2016). Triple-negative breast cancers with amplification of JAK2 at the 9p24 locus demonstrate JAK2-specific dependence. Sci. Transl. Med..

[115] Barrett M.T., Anderson K.S., Lenkiewicz E., Andreozzi M., Cunliffe H.E., Klassen C.L., Dueck A.C., McCullough A.E., Reddy S.K., Ramanathan R.K. (2015). Genomic amplification of 9p24.1 targeting JAK2, PD-L1, and PD-L2 is enriched in high-risk triple negative breast cancer. Oncotarget.

[116] Stanek L., Tesarova P., Vocka M., Petruzelka L. (2018). Analysis of the JAK2 gene in triple-negative breast cancer (TNBC). Ann. Oncol..

[117] Stover D.G., Gil Del Alcazar C.R., Brock J., Guo H., Overmoyer B., Balko J., Xu Q., Bardia A., Tolaney S.M., Gelman R. (2018). Phase II study of ruxolitinib, a selective JAK1/2 inhibitor, in patients with metastatic triple-negative breast cancer. NPJ Breast Cancer.

[118] Johnson D.E., O’Keefe R.A., Grandis J.R. (2018). Targeting the IL-6/JAK/STAT3 signalling axis in cancer. Nat. Rev. Clin. Oncol..

[119] Huynh J., Chand A., Gough D., Ernst M. (2019). Therapeutically exploiting STAT3 activity in cancer—Using tissue repair as a road map. Nat. Rev. Cancer.

[120] Qin J.J., Yan L., Zhang J., Zhang W.D. (2019). STAT3 as a potential therapeutic target in triple negative breast cancer: A systematic review. J. Exp. Clin. Cancer Res..

[121] Morris P.G., Rota S., Cadoo K., Zamora S., Patil S., D’Andrea G., Gilewski T., Bromberg J., Dang C., Dickler M. (2018). Phase II study of paclitaxel and dasatinib in metastatic breast cancer. Clin. Breast Cancer.

[122] Furtek S.L., Backos D.S., Matheson C.J., Reigan P. (2016). Strategies and Approaches of Targeting STAT3 for Cancer Treatment. ACS Chem. Biol..

[123] Xiong A., Yang Z., Shen Y., Zhou J., Shen Q. (2014). Transcription factor STAT3 as a novel molecular target for cancer prevention. Cancers.

[124] Park S.-Y., Choi J.-H., Nam J.-S. (2019). Targeting cancer stem cells in triple-negative breast cancer. Cancers.

[125] Takebe N., Miele L., Harris P.J., Jeong W., Bando H., Kahn M., Yang S.X., Ivy S.P. (2015). Targeting Notch, Hedgehog, and Wnt pathways in cancer stem cells: Clinical update. Nat. Rev. Clin. Oncol..

[126] Solzak J.P., Atale R.V., Hancock B.A., Sinn A.L., Pollok K.E., Jones D.R., Radovich M. (2017). Dual PI3K and Wnt pathway inhibition is a synergistic combination against triple negative breast cancer. NPJ Breast Cancer.

[127] Liu J., Pan S., Hsieh M.H., Ng N., Sun F., Wang T., Kasibhatla S., Schuller A.G., Li A.G., Cheng D. (2013). Targeting Wnt-driven cancer through the inhibition of Porcupine by LGK974. Proc. Natl. Acad. Sci. USA.

[128] Jang G.B., Hong I.S., Kim R.J., Lee S.Y., Park S.J., Lee E.S., Park J.H., Yun C.H., Chung J.U., Lee K.J. (2015). Wnt/β-catenin small-molecule inhibitor CWP232228 preferentially inhibits the growth of breast cancer stem-like cells. Cancer Res..

[129] Gurney A., Axelrod F., Bond C.J., Cain J., Chartier C., Donigan L., Fischer M., Chaudhari A., Ji M., Kapoun A.M. (2012). Wnt pathway inhibition via the targeting of Frizzled receptors results in decreased growth and tumorigenicity of human tumors. Proc. Natl. Acad. Sci. USA.

[130] Fischer M.M., Yen W.-C., Zheng C., Henner R., Cattaruzza F., Tang T., Yeung P., Biswas T., Lewicki J., Gurney A. (2015). Abstract 4233: Wnt pathway antagonist ipafricept (FZD8-Fc, OMP-54F28) inhibits tumor growth and reduces tumor-initiating cell frequency in ovarian patient-derived xenograft models. Cancer Res..

[131] Blagodatski A., Poteryaev D., Katanaev V.L. (2014). Targeting the Wnt pathways for therapies. Mol. Cell. Ther..

[132] Li Y.W., Xu J., Zhu G.Y., Huang Z.J., Lu Y., Li X.Q., Wang N., Zhang F.X. (2018). Apigenin suppresses the stem cell-like properties of triple-negative breast cancer cells by inhibiting YAP/TAZ activity. Cell Death Discov..

[133] Curigliano G., Pivot X., Cortés J., Elias A., Cesari R., Khosravan R., Collier M., Huang X., Cataruozolo P.E., Kern K.A. (2013). Randomized phase II study of sunitinib versus standard of care forpatients with previously treated advanced triple-negative breastcancer. Breast.

[134] Ribatti D., Nico B., Ruggieri S., Tamma R., Simone G., Mangia A. (2016). Angiogenesis and antiangiogenesis in triple-negative breast cancer. Transl. Oncol..

[135] Bender R.J., Mac Gabhann F. (2013). Expression of VEGF and Semaphorin Genes Define Subgroups of Triple Negative Breast Cancer. PLoS ONE.

[136] Bahnassy A., Mohanad M., Ismail M.F., Shaarawy S., El-Bastawisy A., Zekri A.R.N. (2015). Molecular biomarkers for prediction of response to treatment and survival in triple negative breast cancer patients from Egypt. Exp. Mol. Pathol..

[137] Linderholm B.K., Hellborg H., Johansson U., Elmberger G., Skoog L., Lehtiö J., Lewensohn R. (2009). Significantly higher levels of vascular endothelial growth factor (VEGF) and shorter survival times for patients with primary operable triple-negative breast cancer. Ann. Oncol..

[138] Changavi A.A., Shashikala A., Ramji A.S. (2015). Epidermal Growth Factor Receptor Expression in Triple Negative and Nontriple Negative Breast Carcinomas. J. Lab. Physicians.

[139] Rimawi M.F., Shetty P.B., Weiss H.L., Schiff R., Osborne C.K., Chamness G.C., Elledge R.M. (2010). Epidermal growth factor receptor expression in breast cancer association with biologic phenotype and clinical outcomes. Cancer.

[140] Masuda H., Zhang D., Bartholomeusz C., Doihara H., Hortobagyi G.N., Ueno N.T. (2012). Role of epidermal growth factor receptor in breast cancer. Breast Cancer Res. Treat..

[141] Cetin I., Topcul M. (2014). Triple negative breast cancer. Asian Pac. J. Cancer Prev..

[142] Zakaria Z., Zulkifle M.F., Hasan W.A.N.W., Azhari A.K., Raub S.H.A., Eswaran J., Soundararajan M., Syed Husain S.N.A. (2019). Epidermal growth factor receptor (EGFR) gene alteration and protein overexpression in Malaysian triple-negative breast cancer (TNBC) cohort. Onco Targets Ther..

[143] Sharpe R., Pearson A., Herrera-Abreu M.T., Johnson D., Mackay A., Welti J.C., Natrajan R., Reynolds A.R., Reis-Filho J.S., Ashworth A. (2011). FGFR signaling promotes the growth of triple-negative and basal-like breast cancer cell lines both in vitro and in vivo. Clin. Cancer Res..

[144] Dienstmann R., Rodon J., Prat A., Perez-Garcia J., Adamo B., Felip E., Cortes J., Iafrate A., Nuciforo P., Tabernero J. (2014). Genomic aberrations in the FGFR pathway: Opportunities for targeted therapies in solid tumors. Ann. Oncol..

[145] Perez-Garcia J., Muñoz-Couselo E., Soberino J., Racca F., Cortes J. (2018). Targeting FGFR pathway in breast cancer. Breast.

[146] Jung S.-Y., Yi J.Y., Kim M.-H., Song K.-H., Kang S.-M., Ahn J., Hwang S.-G., Nam K.-Y., Song J.-Y. (2015). IM-412 inhibits the invasion of human breast carcinoma cells by blocking FGFR-mediated signaling. Oncol. Rep..

[147] Goldenberg D.M., Cardillo T.M., Govindan S.V., Rossi E.A., Sharkey R.M. (2015). Trop-2 is a novel target for solid cancer therapy with sacituzumab govitecan (IMMU-132), an antibody-drug conjugate (ADC). Oncotarget.

[148] Goldenberg D.M., Stein R., Sharkey R.M. (2018). The emergence of trophoblast cell-surface antigen 2 (TROP-2) as a novel cancer target. Oncotarget.

[149] Sharkey R.M., McBride W.J., Cardillo T.M., Govindan S.V., Wang Y., Rossi E.A., Chang C.H., Goldenberg D.M. (2015). Enhanced delivery of SN-38 to human tumor xenografts with an anti-Trop-2-SN-38 antibody conjugate (sacituzumab govitecan). Clin. Cancer Res..

[150] Starodub A.N., Ocean A.J., Shah M.A., Guarino M.J., Picozzi V.J., Vahdat L.T., Thomas S.S., Govindan S.V., Maliakal P.P., Wegener W.A. (2015). First-in-human trial of a novel anti-trop-2 Antibody-SN-38 conjugate, sacituzumab govitecan, for the treatment of diverse metastatic solid tumors. Clin. Cancer Res..

[151] Bendell J., Saleh M., Rose A.A.N., Siegel P.M., Hart L., Sirpal S., Jones S., Green J., Crowley E., Simantov R. (2014). Phase I/II study of the antibody-drug conjugate glembatumumab vedotin in patients with locally advanced or metastatic breast cancer. J. Clin. Oncol..

[152] Yardley D.A., Weaver R., Melisko M.E., Saleh M.N., Arena F.P., Forero A., Cigler T., Stopeck A., Citrin D., Oliff I. (2015). EMERGE: A randomized phase II study of the antibody-drug conjugate glembatumumab vedotin in advanced glycoprotein NMB - Expressing breast cancer. J. Clin. Oncol..

[153] Marquez-Nostra B.V., Lee S., Laforest R., Vitale L., Nie X., Hyrc K., Keler T., Hawthorne T., Hoog J., Li S. (2017). Preclinical PET imaging of glycoprotein non-metastatic melanoma B in triple negative breast cancer: Feasibility of an antibody-based companion diagnostic agent. Oncotarget.

[154] Liston D.R., Davis M. (2017). Clinically relevant concentrations of anticancer drugs: A guide for nonclinical studies. Clin. Cancer Res..

[155] Lichota A., Gwozdzinski K. (2018). Anticancer activity of natural compounds from plant and marine environment. Int. J. Mol. Sci..

[156] Gao J., Liu Z.J., Chen T., Zhao D. (2014). Pharmaceutical properties of calycosin, the major bioactive isoflavonoid in the dry root extract of Radix astragali. Pharm. Biol..

[157] Massi A., Bortolini O., Ragno D., Bernardi T., Sacchetti G., Tacchini M., de Risi C. (2017). Research progress in the modification of quercetin leading to anticancer agents. Molecules.

[158] Kim J.Y., Dao T.T.P., Song K., Park S.B., Jang H., Park M.K., Gan S.U., Kim Y.S. (2018). Annona muricata Leaf Extract Triggered Intrinsic Apoptotic Pathway to Attenuate Cancerous Features of Triple Negative Breast Cancer MDA-MB-231 Cells. Evid. Based Complement. Alternat. Med..

[159] Lin Y., Shi R., Wang X., Shen H.-M. (2008). Luteolin, a flavonoid with potential for cancer prevention and therapy. Curr. Cancer Drug Targets.

[160] Lin D., Kuang G., Wan J., Zhang X., Li H., Gong X., Li H. (2017). Luteolin suppresses the metastasis of triple-negative breast cancer by reversing epithelial-to-mesenchymal transition via downregulation of β-catenin expression. Oncol. Rep..

[161] Cook M.T., Liang Y., Besch-Williford C., Hyder S.M. (2016). Luteolin inhibits lung metastasis, cell migration, and viability of triple-negative breast cancer cells. Breast Cancer.

[162] Tsai K.J., Tsai H.Y., Tsai C.C., Chen T.Y., Hsieh T.H., Chen C.L., Mbuyisa L., Huang Y.B., Lin M.W. (2021). Luteolin Inhibits Breast Cancer Stemness and Enhances Chemosensitivity through the Nrf2-Mediated Pathway. Molecules.

[163] Ibrahim M.Y., Hashim N.M., Mariod A.A., Mohan S., Abdulla M.A., Abdelwahab S.I., Arbab I.A. (2016). α-Mangostin from Garcinia mangostana Linn: An updated review of its pharmacological properties. Arab. J. Chem..

[164] Scolamiero G., Pazzini C., Bonafè F., Guarnieri C., Muscari C. (2018). Effects of α-mangostin on viability, growth and cohesion of multicellular spheroids derived from human breast cancer cell lines. Int. J. Med. Sci..

[165] Zhu X., Li J., Ning H., Yuan Z., Zhong Y., Wu S., Zeng J.Z. (2021). α-Mangostin Induces Apoptosis and Inhibits Metastasis of Breast Cancer Cells via Regulating RXRα-AKT Signaling Pathway. Front. Pharmacol..

[166] Meghwal M., Goswami T. (2013). Piper nigrum and piperine: An update. Phytother. Res..

[167] Greenshields A.L., Doucette C.D., Sutton K.M., Madera L., Annan H., Yaffe P.B., Knickle A.F., Dong Z., Hoskin D.W. (2015). Piperine inhibits the growth and motility of triple-negative breast cancer cells. Cancer Lett..

[168] Byun H.J., Darvin P., Kang D.Y., Sp N., Joung Y.H., Park J.H., Kim S.J., Yang Y.M. (2017). Silibinin downregulates MMP2 expression via Jak2/STAT3 pathway and inhibits the migration and invasive potential in MDA-MB-231 cells. Oncol. Rep..

[169] Salehi B., Venditti A., Sharifi-Rad M., Kręgiel D., Sharifi-Rad J., Durazzo A., Lucarini M., Santini A., Souto E.B., Novellino E. (2019). The therapeutic potential of apigenin. Int. J. Mol. Sci..

[170] Bauer D., Mazzio E., Hilliard A., Oriaku E.T., Soliman K.F.A. (2020). Effect of apigenin on whole transcriptome profile of TNFα-activated MDA-MB-468 triple negative breast cancer cells. Oncol. Lett..

[171] Nguyen L.T., Lee Y.-H., Sharma A.R., Park J.-B., Jagga S., Sharma G., Lee S.-S., Nam J.-S. (2017). Quercetin induces apoptosis and cell cycle arrest in triple-negative breast cancer cells through modulation of Foxo3a activity. Korean J. Physiol. Pharmacol..

[172] Srinivasan A., Thangavel C., Liu Y., Shoyele S., Den R.B., Selvakumar P., Lakshmikuttyamma A. (2016). Quercetin regulates β-catenin signaling and reduces the migration of triple negative breast cancer. Mol. Carcinog..

[173] Sultan A., Khalil M., Abdelghany B., Alkhuriji A., Sadek O. (2017). Quercetin induces apoptosis in triple-negative breast cancer cells via inhibiting fatty acid synthase and ß-catenin. Int. J. Clin. Exp. Pathol..

[174] Khan N., Syed D.N., Ahmad N., Mukhtar H. (2013). Fisetin: A dietary antioxidant for health promotion. Antioxid. Redox Signal..

[175] Li J., Gong X., Jiang R., Lin D., Zhou T., Zhang A., Li H., Zhang X., Wan J., Kuang G. (2018). Fisetin inhibited growth and metastasis of triple-negative breast cancer by reversing epithelial-to-mesenchymal transition via PTEN/Akt/GSK3β signal pathway. Front. Pharmacol..

[176] Liang Z.-J., Wan Y., Zhu D.-D., Wang M.-X., Jiang H.-M., Huang D.-L., Luo L.-F., Chen M.-J., Yang W.-P., Li H.-M. (2021). Resveratrol Mediates the Apoptosis of Triple Negative Breast Cancer Cells by Reducing POLD1 Expression. Front. Oncol..

[177] Yang M.D., Sun Y., Zhou W.J., Xie X.Z., Zhou Q.M., Lu Y.Y., Su S.B. (2021). Resveratrol Enhances Inhibition Effects of Cisplatin on Cell Migration and Invasion and Tumor Growth in Breast Cancer MDA-MB-231 Cell Models In Vivo and In Vitro. Molecules.

[178] Tuli H.S., Tuorkey M.J., Thakral F., Sak K., Kumar M., Sharma A.K., Sharma U., Jain A., Aggarwal V., Bishayee A. (2019). Molecular mechanisms of action of genistein in cancer: Recent advances. Front. Pharmacol..

[179] Pan H., Zhou W., He W., Liu X., Ding Q., Ling L., Zha X., Wang S. (2012). Genistein inhibits MDA-MB-231 triple-negative breast cancer cell growth by inhibiting NF-κB activity via the Notch-1 pathway. Int. J. Mol. Med..

[180] Fang Y., Zhang Q., Wang X., Yang X., Wang X., Huang Z., Jiao Y., Wang J. (2016). Quantitative phosphoproteomics reveals genistein as a modulator of cell cycle and DNA damage response pathways in triple-negative breast cancer cells. Int. J. Oncol..

[181] Martin A.C.B., Fuzer A.M., Becceneri A.B., da Silva J.A., Tomasin R., Denoyer D., Kim S.-H., McIntyre K.A., Pearson H.B., Yeo B. (2017). [10]-gingerol induces apoptosis and inhibits metastatic dissemination of triple negative breast cancer in vivo. Oncotarget.

[182] Joo J.H., Hong S.S., Cho Y.R., Seo D.W. (2016). 10-Gingerol inhibits proliferation and invasion of MDA-MB-231 breast cancer cells through suppression of Akt and p38MAPK activity. Oncol. Rep..

[183] Rozmer Z., Perjési P. (2016). Naturally occurring chalcones and their biological activities. Phytochem. Rev..

[184] Muchtaridi M., Syahidah H.N., Subarnas A., Yusuf M., Bryant S.D., Langer T. (2017). Molecular Docking and 3D-Pharmacophore Modeling to Study the Interactions of Chalcone Derivatives with Estrogen Receptor Alpha. Pharmaceuticals.

[185] Salehi B., Quispe C., Chamkhi I., El Omari N., Balahbib A., Sharifi-Rad J., Bouyahya A., Akram M., Iqbal M., Docea A.O. (2020). Pharmacological Properties of Chalcones: A Review of Preclinical Including Molecular Mechanisms and Clinical Evidence. Front. Pharmacol..

[186] Shrivastava S., Jeengar M.K., Thummuri D., Koval A., Katanaev V.L., Marepally S., Naidu V. (2017). Cardamonin, a chalcone, inhibits human triple negative breast cancer cell invasiveness by downregulation of Wnt/β-catenin signaling cascades and reversal of epithelial-mesenchymal transition. BioFactors.

[187] Wang N., Tan H.-Y., Li L., Yuen M.-F., Feng Y. (2015). Berberine and Coptidis Rhizoma as potential anticancer agents: Recent updates and future perspectives. J. Ethnopharmacol..

[188] Liu D., Meng X., Wu D., Qiu Z., Luo H. (2019). A natural isoquinoline alkaloid with antitumor activity: Studies of the biological activities of berberine. Front. Pharmacol..

[189] El Khalki L., Maire V., Dubois T., Zyad A. (2020). Berberine impairs the survival of triple negative breast cancer cells: Cellular and molecular analyses. Molecules.

[190] Sun X.-D., Liu X.-E., Huang D.-S. (2012). Curcumin induces apoptosis of triple-negative breast cancer cells by inhibition of EGFR expression. Mol. Med. Rep..

[191] Zhou X., Jiao D., Dou M., Zhang W., Lv L., Chen J., Li L., Wang L., Han X. (2020). Curcumin inhibits the growth of triple-negative breast cancer cells by silencing EZH2 and restoring DLC1 expression. J. Cell. Mol. Med..

[192] Cheng T.-C., Sayseng J.O., Tu S.-H., Juan T.-C., Fang C.-L., Liao Y.-C., Chu C.-Y., Chang H.-W., Yen Y., Chen L. (2021). Curcumin-induced antitumor effects on triple-negative breast cancer patient-derived xenograft tumor mice through inhibiting salt-induced kinase-3 protein. J. Food Drug Anal..

[193] Braicu C., Gherman C. (2013). Epigallocatechin gallate induce cell death and apoptosis in triple negative breast cancer cells Hs578T. J. Drug Target..

[194] Zhu J., Jiang Y., Yang X., Wang S., Xie C., Li X., Li Y., Chen Y., Wang X., Meng Y. (2017). Wnt/β-catenin pathway mediates (-)-Epigallocatechin-3-gallate (EGCG) inhibition of lung cancer stem cells. Biochem. Biophys. Res. Commun..

[195] Oh S., Gwak J., Park S., Yang C.S. (2014). Green tea polyphenol EGCG suppresses Wnt/β-catenin signaling by promoting GSK-3β- and PP2A-independent β-catenin phosphorylation/degradation. BioFactors.

[196] Hong O.Y., Noh E.M., Jang H.Y., Lee Y.R., Lee B.K., Jung S.H., Kim J.S., Youn H.J. (2017). Epigallocatechin gallate inhibits the growth of MDA-MB-231 breast cancer cells via inactivation of the β-catenin signaling pathway. Oncol. Lett..

[197] Crous-Masó J., Palomeras S., Relat J., Camó C., Martínez-Garza Ú., Planas M., Feliu L., Puig T. (2018). (-)-Epigallocatechin 3-Gallate Synthetic Analogues Inhibit Fatty Acid Synthase and Show Anticancer Activity in Triple Negative Breast Cancer. Molecules.

[198] Wang L., Li H., Yang S., Ma W., Liu M., Guo S., Zhan J., Zhang H., Tsang S.Y., Zhang Z. (2016). Cyanidin-3-o-glucoside directly binds to ERα36 and inhibits EGFR-positive triple-negative breast cancer. Oncotarget.

[199] Zhou Y., Chen L., Ding D., Li Z., Cheng L., You Q., Zhang S. (2022). Cyanidin-3-O-glucoside inhibits the β-catenin/MGMT pathway by upregulating miR-214-5p to reverse chemotherapy resistance in glioma cells. Sci. Rep..

[200] Cai Y., Zhao B., Liang Q., Zhang Y., Cai J., Li G. (2017). The selective effect of glycyrrhizin and glycyrrhetinic acid on topoisomerase IIα and apoptosis in combination with etoposide on triple negative breast cancer MDA-MB-231 cells. Eur. J. Pharmacol..

[201] Lin S.C., Chu P.Y., Liao W.T., Wu M.Y., Tsui K.H., Lin L.T., Huang C.H., Chen L.L., Li C.J. (2018). Glycyrrhizic acid induces human MDA-MB-231 breast cancer cell death and autophagy via the ROS-mitochondrial pathway. Oncol. Rep..

[202] Zhou W., Fang H., Wu Q., Wang X., Liu R., Li F., Xiao J., Yuan L., Zhou Z., Ma J. (2019). Ilamycin E, a natural product of marine actinomycete, inhibits triple-negative breast cancer partially through ER stress-CHOP-Bcl-2. Int. J. Biol. Sci..

[203] Xu X., Rajamanicham V., Xu S., Liu Z., Yan T., Liang G., Guo G., Zhou H., Wang Y. (2019). Schisandrin A inhibits triple negative breast cancer cells by regulating Wnt/ER stress signaling pathway. Biomed. Pharmacother..

[204] Tieng F.Y.F., Latifah S.Y., Md Hashim N.F., Khaza’ai H., Ahmat N., Gopalsamy B., Wibowo A. (2019). Ampelopsin E reduces the invasiveness of the triple negative breast cancer cell line, MDA-MB-231. Molecules.

[205] Shrestha S., Sorolla A., Fromont J., Blancafort P., Flematti G.R. (2018). Aurantoside c targets and induces apoptosis in triple negative breast cancer cells. Mar. Drugs.

[206] Robles A.J., McCowen S., Cai S., Glassman M., Ruiz F., Cichewicz R.H., McHardy S.F., Mooberry S.L. (2017). Structure-Activity Relationships of New Natural Product-Based Diaryloxazoles with Selective Activity against Androgen Receptor-Positive Breast Cancer Cells. J. Med. Chem..

[207] Jang H., Ko H., Song K., Kim Y.S. (2019). A sesquiterpenoid from farfarae flos induces apoptosis of MDA-MB-231 human breast cancer cells through inhibition of JAK-STAT3 signaling. Biomolecules.

[208] Fatima I., El-Ayachi I., Taotao L., Lillo M.A., Krutilina R., Seagroves T.N., Radaszkiewicz T.W., Hutnan M., Bryja V., Krum S.A. (2017). The natural compound Jatrophone interferes with Wnt/β-catenin signaling and inhibits proliferation and EMT in human triple-negative breast cancer. PLoS ONE.

[209] Ling T., Hadi V., Guiguemde A., Landfear S.M., Rivas F., Jetter R. (2015). Jatropha natural products as potential therapeutic leads. The Formation, Structure and Activity of Phytochemicals. Recent Advances in Phytochemistry.

[210] Li H., Yang B., Huang J., Xiang T., Yin X., Wan J., Luo F., Zhang L., Li H., Ren G. (2013). Naringin inhibits growth potential of human triple-negative breast cancer cells by targeting β-catenin signaling pathway. Toxicol. Lett..

[211] Jaspal B., Norman F., Kayla A., Maria C.T., Bela P. (2018). A novel anti-triple negative breast cancer compound isolated from medicinal herb Myrothamnus flabellifolius. J. Med. Plants Res..

[212] Zhang W., Yu W., Cai G., Zhu J., Zhang C., Li S., Guo J., Yin G., Chen C., Kong L. (2018). A new synthetic derivative of cryptotanshinone KYZ3 as STAT3 inhibitor for triple-negative breast cancer therapy. Cell Death Dis..

[213] Bonaccorsi P.M., Labbozzetta M., Barattucci A., Salerno T.M.G., Poma P., Notarbartolo M. (2019). Synthesis of curcumin derivatives and analysis of their antitumor effects in triple negative breast cancer (TNBC) cell lines. Pharmaceuticals.

[214] Rios-Fuller T.J., Ortiz-Soto G., Lacourt-Ventura M., Maldonado-Martinez G., Cubano L.A., Schneider R.J., Martinez-Montemayor M.M. (2018). Ganoderma lucidum extract (GLE) impairs breast cancer stem cells by targeting the STAT3 pathway. Oncotarget.

[215] Chen Y., Chen Z.y., Chen L., Zhang J.y., Fu L.y., Tao L., Zhang Y., Hu X.x., Shen X.c. (2019). Shikonin inhibits triple-negative breast cancer-cell metastasis by reversing the epithelial-to-mesenchymal transition via glycogen synthase kinase 3β-regulated suppression of β-catenin signaling. Biochem. Pharmacol..

[216] Liu C., Wang K., Zhuang J., Gao C., Li H., Liu L., Feng F., Zhou C., Yao K., Deng L. (2019). The modulatory properties of astragalus membranaceus treatment on triple-negative breast cancer: An integrated pharmacological method. Front. Pharmacol..

[217] Kim D., Wang C.Y., Hu R., Lee J.Y., Luu T.T.T., Park H.J., Lee S.K. (2019). Antitumor Activity of Vanicoside B Isolated from Persicaria dissitiflora by Targeting CDK8 in Triple-Negative Breast Cancer Cells. J. Nat. Prod..

[218] Lou C., Chen Y., Zhang J., Yang B., Zhao H. (2019). Eupalinolide J Suppresses the growth of triple-negative breast cancer cells via targeting STAT3 signaling pathway. Front. Pharmacol..

[219] Li H.C., Xia Z.H., Chen Y.F., Yang F., Feng W., Cai H., Mei Y., Jiang Y.M., Xu K., Feng D.X. (2018). Cantharidin Inhibits the Growth of Triple-Negative Breast Cancer Cells by Suppressing Autophagy and Inducing Apoptosis In Vitro and In Vivo. Cell. Physiol. Biochem..

[220] Gangrade A., Pathak V., Augelli-Szafran C.E., Wei H.X., Oliver P., Suto M., Buchsbaum D.J. (2018). Preferential inhibition of Wnt/β-catenin signaling by novel benzimidazole compounds in triple-negative breast cancer. Int. J. Mol. Sci..

[221] Kong Y., Chen J., Zhou Z., Xia H., Qiu M.H., Chen C. (2014). Cucurbitacin e induces cell cycle G2/M phase arrest and apoptosis in triple negative breast cancer. PLoS ONE.

[222] Huang W., Liang Y., Ma X. (2019). Alpha-mangostin induces endoplasmic reticulum stress and autophagy which count against fatty acid synthase inhibition mediated apoptosis in human breast cancer cells. Cancer Cell Int..

[223] Choi Y.H. (2018). Schisandrin A prevents oxidative stress-induced DNA damage and apoptosis by attenuating ROS generation in C2C12 cells. Biomed. Pharmacother..

[224] Wu C.T., Mulabagal V., Nalawade S.M., Chen C.L., Yang T.F., Tsay H.S. (2003). Isolation and quantitative analysis of cryptotanshinone, an active quinoid diterpene formed in callus of Salvia miltiorrhiza BUNGE. Biol. Pharm. Bull..

[225] Auyeung K.K., Han Q.-B., Ko J.K. (2016). Astragalus membranaceus: A review of its protection against inflammation and gastrointestinal cancers. Am. J. Chin. Med..

[226] Wang F., Zhong H., Fang S., Zheng Y., Li C., Peng G., Shen X. (2018). Potential anti-inflammatory sesquiterpene lactones from Eupatorium lindleyanum. Planta Med..

[227] Tian S., Chen Y., Yang B., Lou C., Zhu R., Zhao Y., Zhao H. (2018). F1012-2 inhibits the growth of triple negative breast cancer through induction of cell cycle arrest, apoptosis, and autophagy. Phytother. Res..

[228] Yan G., Ji L., Luo Y., Hu Y. (2011). Antioxidant activities of extracts and fractions from Eupatorium lindleyanum DC. Molecules.

[229] Liu Y.-P., Li L., Xu L., Dai E.N., Chen W.-D. (2018). Cantharidin suppresses cell growth and migration, and activates autophagy in human non-small cell lung cancer cells. Oncol. Lett..

[230] Newton E.E., Mueller L.E., Treadwell S.M., Morris C.A., Machado H.L. (2022). Molecular Targets of Triple-Negative Breast Cancer: Where Do We Stand?. Cancers.

[231] Cao J., Zhang M., Wang B., Zhang L., Fang M., Zhou F. (2021). Chemoresistance and Metastasis in Breast Cancer Molecular Mechanisms and Novel Clinical Strategies. Front. Oncol..

[232] Zhang Y., Wang X. (2020). Targeting the Wnt/β-catenin signaling pathway in cancer. J. Hematol. Oncol..

[233] Vagia E., Mahalingam D., Cristofanilli M. (2020). The Landscape of Targeted Therapies in TNBC. Cancers.

[234] Wahba H.A., El-Hadaad H.A. (2015). Current approaches in treatment of triple-negative breast cancer. Cancer Biol. Med..

[235] Maqbool M., Bekele F., Fekadu G. (2022). Treatment Strategies Against Triple-Negative Breast Cancer: An Updated Review. Breast Cancer.

